# Targeted Inhibition of the NUP98-NSD1 Fusion Oncogene in Acute Myeloid Leukemia

**DOI:** 10.3390/cancers12102766

**Published:** 2020-09-26

**Authors:** Sagarajit Mohanty, Nidhi Jyotsana, Amit Sharma, Arnold Kloos, Razif Gabdoulline, Basem Othman, Courteney K. Lai, Renate Schottmann, Madhvi Mandhania, Johannes Schmoellerl, Florian Grebien, Euan Ramsay, Anitha Thomas, Hans-Peter Vornlocher, Arnold Ganser, Felicitas Thol, Michael Heuser

**Affiliations:** 1 Department of Hematology, Hemostasis, Oncology and Stem Cell Transplantation, Hannover Medical School, 30625 Hannover, Germany; mohanty.sagarajit@mh-hannover.de (S.M.); starsamit@gmail.com (A.S.); Kloos.Arnold@mh-hannover.de (A.K.); Gabdoulline.Razif@mh-hannover.de (R.G.); Othman.Basem@mh-hannover.de (B.O.); Lai.Courteney@mh-hannover.de (C.K.L.); Schottmann.Renate@mh-hannover.de (R.S.); Ganser.Arnold@mh-hannover.de (A.G.); Thol.Felicitas@mh-hannover.de (F.T.); 2 Department of Biomedical Engineering, Vanderbilt University, Nashville, TN 37235, USA; jyotsana.nidhi@gmail.com; 3 National Centre for Cell Science, Pune 411007, India; madhvi.mandhania@gmail.com; 4 Institute for Medical Biochemistry, University of Veterinary Medicine Vienna, 1210 Vienna, Austria; johannes.schmoellerl@imp.ac.at (J.S.); florian.grebien@vetmeduni.ac.at (F.G.); 5 Precision NanoSystems Inc., Vancouver, BC V6P 6T7, Canada; eramsay@precision-nano.com (E.R.); athomas@precision-nano.com (A.T.); 6 AxolabsGmbH, 95326 Kulmbach, Germany; Hans-Peter.Vornlocher@axolabs.com

**Keywords:** AML, NUP98-NSD1, NRASG12D, siRNA, liposome

## Abstract

**Simple Summary:**

NUP98-NSD1-positive acute myeloid leukemia (AML) frequently shows an additional mutation in Neuroblastoma rat sarcoma (NRAS). However, the synergistic effect of NUP98-NSD1 and NRASG12D in leukemic transformation remained unclear. In addition, NUP98-NSD1 positive AML patients respond poorly to chemotherapy and lack a targeted therapeutic option. Our study aimed to identify the cooperation of NUP98-NSD1 fusion and NRASG12D mutation and to develop a novel therapeutic approach for this AML. We found that NUP98-NSD1 alone can cause leukemia with long latency, and NRASG12D contributes to the aggressiveness of this AML. Additionally, we validated a novel NUP98-NSD1-targeting siRNA/lipid nanoparticle formulation that significantly prolonged the survival of patient-derived xenograft (PDX) mice with NUP98-NSD1-positive AML.

**Abstract:**

NUP98-NSD1-positive acute myeloid leukemia (AML) is a poor prognostic subgroup that is frequently diagnosed in pediatric cytogenetically normal AML. NUP98-NSD1-positive AML often carries additional mutations in genes including *FLT3, NRAS, WT1,* and *MYC*. The purpose of our study was to characterize the cooperative potential of the fusion and its associated Neuroblastoma rat sarcoma (NRAS) mutation. By constitutively expressing NUP98-NSD1 and NRASG12D in a syngeneic mouse model and using a patient-derived xenograft (PDX) model from a NUP98-NSD1-positive AML patient, we evaluated the functional role of these genes and tested a novel siRNA formulation that inhibits the oncogenic driver NUP98-NSD1. NUP98-NSD1 transformed murine bone marrow (BM) cells in vitro and induced AML in vivo. While NRASG12D expression was insufficient to transform cells alone, co-expression of NUP98-NSD1 and NRASG12D enhanced the leukemogenicity of NUP98-NSD1. We developed a NUP98-NSD1-targeting siRNA/lipid nanoparticle formulation that significantly prolonged the survival of the PDX mice. Our study demonstrates that mutated NRAS cooperates with NUP98-NSD1 and shows that direct targeting of the fusion can be exploited as a novel treatment strategy in NUP98-NSD1-positive AML patients.

## 1. Introduction

Acute myeloid leukemia (AML) is a heterogeneous disease of myeloid cells, which is characterized by many different genetic aberrations and requires an increasingly complex approach to treatment [1,2]. Among these aberrations, fusion genes have been frequently and consistently reported [3].

The *Nucleoporin 98 (NUP98*) gene, located on chromosome 11, has many different fusion partners in different hematological malignancies including both homeobox (*HOXA9*, *HOXC11*, *HOXD13*) and non-homeobox genes (*JAR1D1A*, *DDX10*, *TOP1*, and *NSD1*) [4]. Among its most frequent fusion partners is the nuclear receptor-binding SET domain protein 1 (NSD1) [5]. Patients are usually diagnosed with normal karyotype AML (CN-AML) and a high white blood cell (WBC) count [6,7]. Although the frequency of NUP98-NSD1 AML is low in the adult CN-AML cohort (2.3%), it is more prevalent (16.1%) in pediatric CN-AML patients [6]. NUP98-NSD1 leukemia frequently co-occurs with mutations in *FLT3*, *NRAS*, *WT1*, and *MYC* [6,7,8,9] and is associated with poor prognosis [6,10,11], with patients showing a poor response to conventional therapy such as chemotherapy and allogeneic hematopoietic stem cell transplantation (allo-HCT). Additionally, Wang et al. reported that some NUP98-NSD1 patients do not achieve remission after chemotherapy and some show early relapse after chemotherapy, underscoring the treatment challenges facing these patients [12].

NUP98-NSD1 occurs as the cryptic translocation t (5; 11) (q35.2; p15.4) [13]. The N-terminal fusion partner NUP98 plays an important role in the nucleo-cytoplasmic exchange of macromolecules across the nuclear membrane [14]. Phe-Gly (FG) repeats of NUP98 are retained in NUP98 fusions and are required for recruiting the CBP/p300 acetyltransferase for histone modification [15]. The C-terminal fusion partner NSD1 is a member of the family of histone methyltransferases, which is encoded by its SET domain [16]. Mutations in the C-terminal partners of NUP98 fusions like NUP98-NSD1, NUP98-JARID1A, NUP98-DDX10, and NUP98-HOXD13 abrogated the leukemic transformation induced by these fusion proteins, indicating the importance of targeting the functional properties of the C-terminal partner gene to treat these leukemias [15,17,18,19].

Significant evidence ties *HOX* gene expression to NUP98-NSD1. Microarray studies have identified a positive correlation of *HOX* cluster genes in NUP98-NSD1-positive AML patients [6]. Additionally, Wang et al. demonstrated that constitutive expression of NUP98-NSD1 in mouse BM cells upregulates distal *Hoxa* cluster gene expression though H3K36 methylation catalyzed by NSD1 SET domain [15]. However, the role of the driver fusion oncogene and the impact of its inhibition in leukemic development in mice have been poorly studied. Additionally, the development of patient-derived xenograft (PDX) models is essential to understand the genetic complexity of human leukemia and improve the preclinical evaluation of anticancer drugs [20]. Here, we investigated the cooperative potential of the oncogenic NUP98-NSD1 and its associated mutation *NRASG12D* in vitro and in vivo. To better understand the pathophysiology of NUP98-NSD1, we developed a patient-derived xenograft (PDX) model by transplanting primary patient cells into immunodeficient mice and demonstrated that targeted inhibition of the driver oncogene NUP98-NSD1 provides a survival advantage in this type of AML.

## 2. Materials and Methods

### 2.1. Cloning

The NUP98-NSD1 plasmid was received as a gift from the Institute for Medical Biochemistry, University of Veterinary Medicine Vienna, Austria. It was cloned in the retroviral expression vector MSCV-IRES-GFP. The NRASG12D plasmid was obtained from ADDGENE (Plasmid #83176) and cloned in the retroviral expression vector MSCV-IRES-BFP. For siRNA screening for both major and minor clone, 500 base pairs across the junction were cloned in the MSCV-IRES-GFP vector. The cloned sequences were confirmed using Sanger sequencing.

### 2.2. Mice and Retroviral Infections of Primary Mouse Bone Marrow Cells

Six to eight-week-old female C57BL/6J mice were obtained from Charles River (Sulzfeld, Germany) and 6–8-week-old female NOD.Cg-Prkdcscid Il2rgtm1Wjl/SzJ (NSG) mice were purchased from Hannover Medical School (Hannover, Germany). Mice were kept in pathogen-free conditions at the central animal laboratory of Hannover Medical School. All animal experiments were performed with permission of the Lower Saxony State Office for Consumer Protection (Oldenburg, Germany) and approval of the ethics committee of Hannover Medical Schhol (vote 2504-2014). To enrich for immature murine bone marrow cells, donor mice were treated with 5-fluorouracil (5-FU) at 150 mg/kg. Treatment with 5-FU inhibits most of the dividing and thus differentiated cells, while it spares the non-dividing immature stem cell population. This ensures that the bone marrow is enriched in stem and progenitor cells for an efficient transduction with retroviral vectors. Four days post-treatment, primary mouse bone marrow cells were harvested and pre-stimulated for 48 h in Dulbecco’s modified Eagle medium (DMEM) (STEM cell Technologies, Cologne, Germany) supplemented with 15% fetal bovine serum (FBS) (Sigma-Aldrich, Munich, Germany), 10 ng/mL human interleukin-6 (hIL-6), 6 ng/mL murine interleukin-3 (mIL-3), and 20 ng/mL murine stem cell factor (mSCF; all from PeproTech, Hamburg, Germany). Retrovirus-producing Phoenix packaging cells were transfected with the required vectors and viral supernatant was used to transduce mouse bone marrow cells using 5 μg/mL protamine sulfate (Sigma-Aldrich, Seelze, Germany). After cells were expanded, they were sorted for the respective fluorochrome expression. Details of the transduction method were described previously [21]. Details on mouse transplantation and treatment can be found in the Supplementary Methods.

### 2.3. Proliferation Assay

Bone marrow from 5-fluorouracil-treated C57BL6/J mice was harvested and transduced with NUP98-NSD1-GFP and NRASG12D-BFP vectors. Four days after transduction, cells were assessed for NRAS-BFP+, NUP98-NSD1-GFP+, and NUP98-NSD1+NRAS-GFP+BFP+ population expression by flow cytometry. Cells were grown in Dulbecco’s modified Eagle medium (DMEM) (STEM cell Technologies, Cologne, Germany) supplemented with 15% fetal bovine serum (FBS) (Sigma-Aldrich, Munich, Germany), 10 ng/mL human interleukin-6 (hIL-6), 6 ng/mL murine interleukin-3 (mIL-3), and 20 ng/mL murine stem cell factor (mSCF; all from PeproTech, Hamburg, Germany). Changes in fluorochrome expression in NRAS-BFP+, NUP98-NSD1-GFP+, and NUP98-NSD1+NRAS -GFP+BFP+ cells were monitored until day 12 of in vitro culture using the BD LSRII flow cytometer (BD Biosciences, Heidelberg, Germany).

### 2.4. Apoptosis Assay

To quantify apoptosis, 1 × 10^5^ cells were stained with Annexin V-APC according to the manufacturer’s protocol (BD Pharmingen; catalog no. 550474). Annexin V was tested at different time points until 12 days after transduction. Fluorescence was measured using a BD LSRII flow cytometer (BD Biosciences, Heidelberg, Germany).

### 2.5. Lipid Nanoparticle (LNPs)/siRNA Formulation

The lipid mixture that we used to package our target siRNAs contained a proprietary lipid mix (Precision Nanosystems, Vancouver, BC, Canada, with minor modifications). Details of the LNP/siRNA formulation and quantification can be found in the Supplementary Methods.

### 2.6. RNA Extraction, cDNA Synthesis, and Quantitative RT-PCR

Total RNA was extracted from cells with the RNeasy kit (Qiagen, Düsseldorf, Germany), according to the manufacturer’s protocol. cDNA was prepared using a Reverse Transcription Kit (Thermo Fisher Scientific GmbH, Bremen, Germany), according to the manufacturer’s instructions.

Quantitative reverse-transcriptase (RT)-PCR was done as previously described using SYBR green (Qiagen) for quantification of double-stranded DNA on a StepOne Plus cycler (Thermo Fisher Scientific) [22]. Relative expression was measured using the 2^−∆∆CT^ method, and the housekeeping gene transcript Abl1 was used to normalize the results. The primer sequences are listed in Table S1.

### 2.7. Gene Expression Profiling and Analysis

NRAS-BFP+, NUP98-NSD1-GFP+, and NUP98-NSD1+NRAS-GFP+BFP+ cells were sorted and expanded after transduction, and RNA was isolated as described above. RNA samples were processed at the Helmholtz Center for Infection Research (Braunschweig, Germany) and sequenced using paired end read 50 bp chemistry on a Nova seq 6000 instrument (Illumina, San Diego, CA, USA). RNAseq sequences were aligned to mouse genome GRCm38 with the help of Gencode v. m22 [Gencode] [23] transcriptome annotations. The main alignment program was STAR [STAR] [24]; Bowtie2 [bowtie2] [25] was used for comparison. Mapping to the transcriptome and gene expression analysis was done with TopHat and Cufflinks [TopHat] [26] with the help of Gencode annotation of genes and alternative transcript variants. Gene and transcript expression was quantified by FPKM (fragments per kilobase per million), TPM (transcripts per million) values, as well as by fragment counts. The latter is calculated from cufflinks outputs using the program HTSeq [HTSeq] [27] and used as input to the DESeq2 [DESeq2] analysis [28]. The Broad Institute Gene Set Enrichment Analysis (GSEA) software was used for gene set enrichment analysis using different gene ontology datasets from the molecular signature database (http://www.broad.mit.edu/gsea/msigdb/). The David online tool was used for pathway enrichment analysis (https://david.ncifcrf.gov/). RNAseq data are available at Gene Expression Omnibus (GEO) under accession number GSE152244 (https://www.ncbi.nlm.nih.gov/geo/query/acc.cgi?acc=GSE152244).

### 2.8. Molecular Mutation Analysis

DNA sequencing libraries were prepared from samples at diagnosis and after each transplantation with a custom TruSight myeloid sequencing panel according to the manufacturers’ instructions (Illumina, San Diego, CA, USA), which included 46 entire genes or hotspots recurrently found in myeloid leukemias (Table S1). Sequencing and sequencing analysis was performed as previously described [29]. The fusion gene was amplified using PCR and sequenced by Sanger sequencing to confirm the fusion break point. The minor subclone of the fusion gene was detected using the mutation surveyor software (Softgenetics, State College, PA, USA).

### 2.9. Statistical Analysis

Data are presented as mean ± standard error of mean (SEM). Pairwise comparisons were performed using the Student’s *t*-test for continuous variables. The log rank test was calculated to compare the survival difference between two groups and Kaplan–Meier curves were used to visualize the results. Statistical significance was set at two-sided *p* < 0.05. All statistical analyses were carried out with GraphPad Prism Version 7 software (GraphPad Software, La Jolla, CA, USA). Graphs were prepared using Adobe Illustrator CS5.1 (Adobe Systems GmbH, Munich, Germany).

## 3. Results

### 3.1. NUP98-NSD1 Immortalizes Murine Hematopoietic Cells and Cooperates with Mutated NRASG12D In Vitro

**Figure 1:**
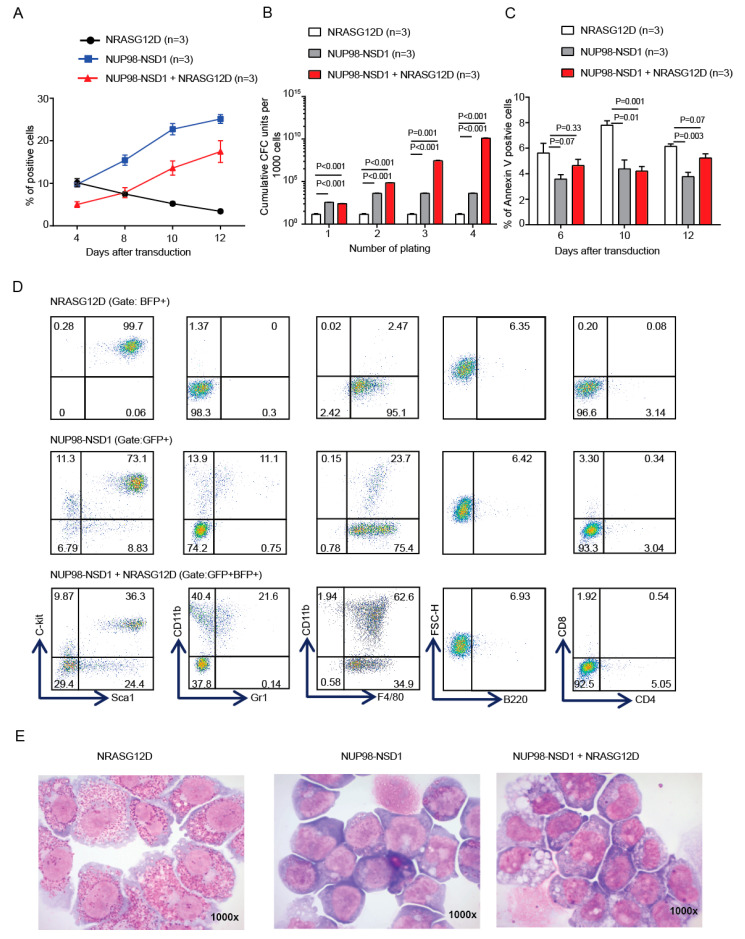
NUP98-NSD1 immortalizes murine hematopoietic cells and cooperates with mutated NRASG12D in vitro. (**A**) Percentage of 5-fluorouracil (5-FU)-treated bone marrow cells that were transduced with either NRASG12D-BFP, NUP98-NSD1-GFP, or both NUP98-NSD1-GFP and NRASG12D-BFP from day 4 to day 12 after transduction (mean ± SEM). (**B**) Cumulative Colony-forming cell unit (CFU) yield is shown for an initial plating of 1000 NRASG12D, NUP98-NSD1, or NRASG12D+NUP98-NSD1 transduced cells (mean ± SEM). (**C**) Percentage of apoptotic cells transduced with NRASG12D, NUP98-NSD1, or NRASG12D+NUP98-NSD1 until 12 days after transduction (mean ± SEM). (**D**) Representative immunophenotype of cells transduced with NRASG12D, NUP98-NSD1, or NRASG12D+NUP98-NSD1. (**E**) Representative Wright–Giemsa–stained cytospin preparations of NRASG12D, NUP98-NSD1, and NUP98-NSD1+NRASG12D transduced mouse bone marrow cells (1000×).

NUP98-NSD1-positive AML is often associated with other mutations like *FLT3-ITD, NRASG12D, or MYC*. To understand the cooperative potential of this fusion gene and its associated mutations in the development and progression of leukemia, we cloned NUP98-NSD1 into the MSCV-IRES-GFP vector and NRASG12D into the MSCV-IRES-BFP vector, respectively, and transduced 5-fluorouracil-treated mouse bone marrow cells (Figure S1A). NUP98-NSD1 and NUP98-NSD1+NRASG12D-expressing cells proliferated in vitro from day 4 to day 12 after transduction and had a significantly higher clonogenic potential in colony-forming cell unit (CFU) assays, while NRASG12D-expressing cell numbers declined in culture and formed no colonies after the first plating in CFU assays (Figure 1A,B). Moreover, there was a modest increase in the proportion of apoptotic cells in NRASG12D compared to NUP98-NSD1 and NUP98-NSD1+NRASG12D-expressing cells 10 days after transduction (Figure 1C). Immunophenotypic and morphologic analyses showed that NUP98-NSD1+NRASG12D cells were comprised of higher proportions of CD11b+/Gr1- immature myeloid cells compared to NUP98-NSD1 cells (Figure 1D,E, Figure S1B). In contrast, NRASG12D cells were predominantly IgER+ mast cells (Figure 1E, Figure S1B,C). Thus, NRASG12D alone is unable to transform bone marrow, while the fusion NUP98-NSD1 can immortalize bone marrow and cooperates with NRASG12D to induce a more immature cell phenotype.

### 3.2. NUP98-NSD1 Induces a Long-Latency AML and Cooperates with NRASG12D to Induce an Aggressive AML In Vivo

**Figure 2:**
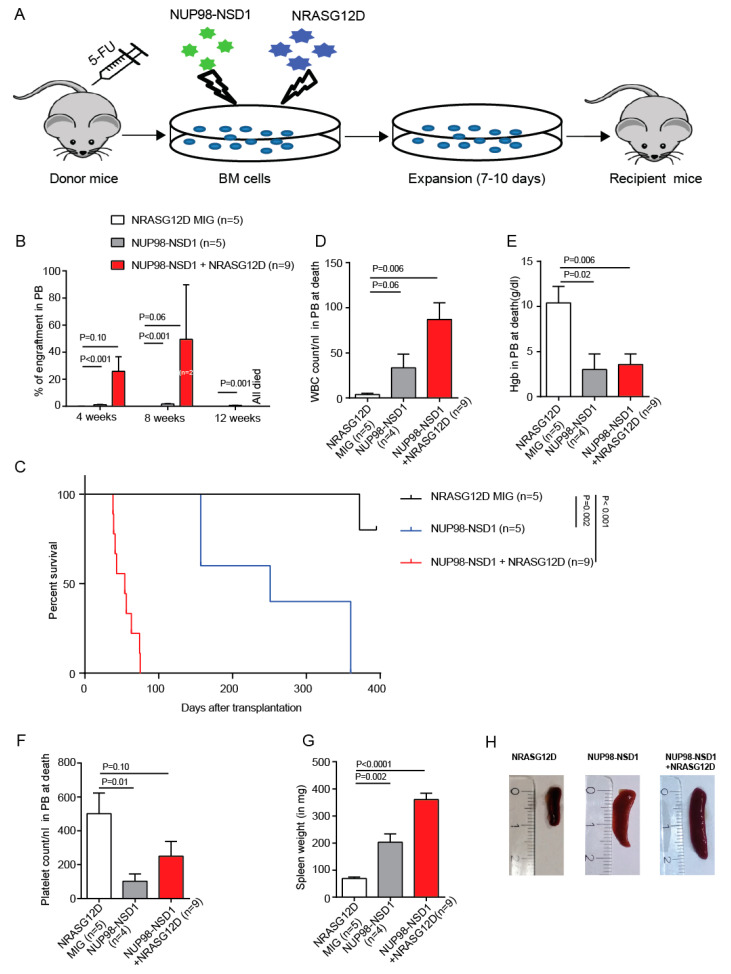
NUP98-NSD1 induces a long-latency AML and cooperates with NRASG12D to induce an aggressive acute myeloid leukemia in vivo. (**A**) Development of syngeneic mouse models using constitutively expressed NRASG12D, NUP98-NSD1, or both. (**B**) Engraftment of transduced cells in peripheral blood of the indicated groups 4, 8, and 12 weeks after transplantation (mean ± SEM). (**C**) Survival of mice that received transplants of cells transduced with the indicated constructs. (**D**) White blood cell count in peripheral blood at time of sacrifice from mice, which received transplants of the indicated cells (mean ± SEM). (**E**) Hemoglobin levels in peripheral blood at time of sacrifice of mice, which received transplants of the indicated cells (mean ± SEM). (**F**) Platelet count in peripheral blood at time of sacrifice of mice, which received transplants of the indicated cells (mean ± SEM). (**G**) Average spleen weight in mice at time of sacrifice of mice, which received transplants of the indicated cells (mean ± SEM). (**H**) Representative image of a spleen at time of sacrifice.

To assess the leukemic potential of NRASG12D, NUP98-NSD1, and NUP98-NSD1+NRASG12D in vivo, we transplanted equal numbers of sorted transduced mouse bone marrow cells in mice (Figure 2A). Mice transplanted with NRASG12D transduced cells did not show any engraftment over 11 months (Figure 2B). NUP98-NSD1 mice showed very low engraftment, while NUP98-NSD1+NRASG12D mice showed rapidly increasing engraftment over 12 weeks (Figure 2B), accompanied by an increased WBC count, anemia, and thrombocytopenia at 8 weeks after transplantation (Figure S2A–C). Mice transplanted with NUP98-NSD1-expressing cells developed leukemia with long latency and mice transplanted with NUP98-NSD1+NRASG12D-expressing cells developed aggressive leukemia with short latency (median survival 251 vs. 54 days after transplantation, *p* = 0.001; Figure 2C). Moribund mice transplanted with NUP98-NSD1 or NUP98-NSD1+NRASG12D showed increased white blood cell counts and reduced hemoglobin and platelet counts (Figure 2D–F) and splenomegaly at time of sacrifice (Figure 2G,H). Morphology and immunophenotype confirmed leukemia in NUP98-NSD1 and NUP98-NSD1+NRASG12D-expressing cells at time of sacrifice in bone marrow, spleen, and peripheral blood (Figures S2D–F and S3A–D). Secondary transplantation of both NUP98-NSD1 and NUP98-NSD1+NRASG12D-expressing cells from primary transplants resulted in leukemia with very short latency compared to the primary transplants (Figure S4A). At time of sacrifice, bone marrow from these mice showed high engraftment, increased WBC counts, and enlarged spleens at death, and NUP98-NSD1+NRASG12D mice showed a higher proliferative potential compared to NUP98-NSD1 mice (Figure S4B–E). These data suggest that NUP98-NSD1 is the leukemic driver, while mutated NRAS cooperates with NUP98-NSD1 and accelerates leukemic onset.

### 3.3. Common and Distinct Pathways Are Regulated by NUP98-NSD1 and NRASG12D

**Figure 3:**
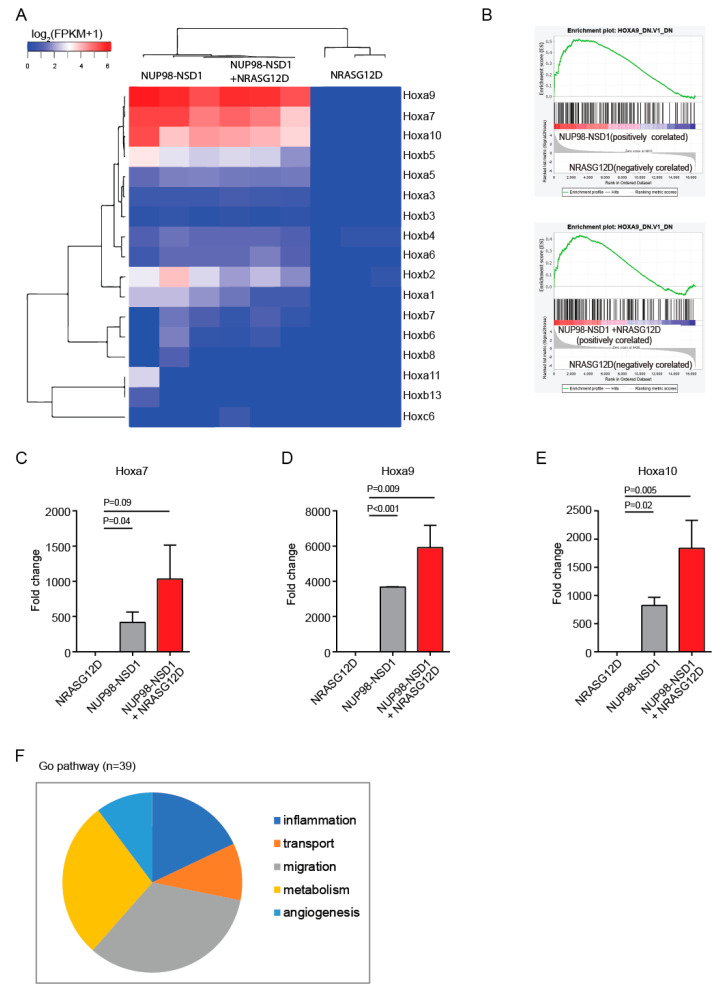
Common and distinct pathways are regulated by NUP98-NSD1 and NRASG12D. (**A**) Heat map from hierarchical clustering showing differential expression of HOX genes in NRASG12D, NUP98-NSD1, and NUP98-NSD1 + NRASG12D transformed cells. (**B**) Enrichment plot for the gene sets HOXA9_DN.V1_DN of Gene Set Enrichment Analysis (GSEA) comparing NUP98-NSD1 and NRASG12D transformed cells. (**C**) Quantitative RT-PCR of Hoxa7 in NRASG12D, NUP98-NSD1, and NUP98-NSD1+NRASG12D transformed mouse bone marrow cells (mean ± SEM). (**D**) Quantitative RT-PCR of Hoxa9 in NRASG12D, NUP98-NSD1, and NUP98-NSD1+NRASG12D transformed mouse bone marrow cells (mean ± SEM). (**E**) Quantitative RT-PCR of Hoxa10 in NRASG12D, NUP98-NSD1, and NUP98-NSD1+NRASG12D transformed mouse bone marrow cells (mean ± SEM). (**F**) Pie chart showing the top 39 biological categories regulated by NRASG12D from the GO biological process pathway gene set analysis comparing NUP98-NSD1+NRASG12D with NUP98-NSD1.

To identify target genes of NUP98-NSD1, we performed gene expression profiling of NUP98-NSD1, NRASG12D, and NUP98-NSD1+NRASG12Dexpressing cells. NUP98-NSD1 and NUP98-NSD1+NRASG12D transformed cells showed a differential gene expression pattern compared to NRASG12D transformed cells (Figure S5A and Tables S4 and S5). Principal component analysis showed distinct overall gene expression levels in NUP98-NSD1 (F), NRASG12D (M), and the combination of NUP98-NSD1/NRASG12D (FM) cells, suggesting that combined expression of the oncogenes modifies the cellular identity (Figure S5B). Importantly, both NUP98-NSD1 and NUP98-NSD1+NRASG12D cells had a distinct *Hox* gene signature characterized by strong upregulation of *Hoxa7*, *Hoxa9*, and *Hoxa10* compared to NRASG12D cells (Figure 3A). Additionally, the Hoxa9 pathway was strongly upregulated in NUP98-NSD1 and NUP98-NSD1+NRASG12D compared to NRASG12D cells (Figure 3B). Quantitative RT-PCR analysis confirmed a significant increase in *Hoxa7*, *Hoxa9,* and *Hoxa10* expression in NUP98-NSD1 and NUP98-NSD1+NRASG12D cells (Figure 3C–E). Distal Hox gene expression is involved in regulating self-renewal of hematopoietic progenitor cells [4,30], confirming that these genes maintain the immature leukemic population in NUP98-NSD1 leukemia through Hox upregulation.

NUP98-NSD1 transformed cells were highly enriched for the gene sets acute myeloid leukemia, hematopoietic cell lineage, and Cyclin D1 when compared to NRASG12D cells (Figure S6A–C). To explore the contribution of NRASG12D to the enhanced proliferative potential of NUP98-NSD1+NRASG12D cells, we compared their gene expression profile to NUP98-NSD1 cells. In addition to mitogen-activated protein kinase 13 signaling (MAPK13), NRASG12D contributed to elevated expression of cyclin D1 (Ccnd1) and different cystatin family genes (Cstdc5, Cstdc4, Csta2, Csta3, Cstdc6), which have been implicated in malignant progression and metastasis (Table S6) [31]. S100 calcium binding protein A8 and A9 (S100a8 and S100a9) genes were upregulated in NUP98-NSD1 transformed cells, and their expression was further enhanced by the addition of NRASG12D (Tables S4 and S6). In line with S100a8 and S100a9 upregulation, gene ontology biological process analysis showed that NRASG12D contributed to different biological processes such as inflammation, transport, migration, metabolism, and angiogenesis (Figure 3F and Table S7). These analyses suggest that upregulation of Hox genes by NUP98-NSD1 is enhanced by NRASG12D that induces a more aggressive phenotype through activation of inflammation, transport, migration, and metabolism pathways.

### 3.4. Establishment and Characterization of a NUP98-NSD1 Patient-Derived Xenograft (PDX) Model

**Figure 4:**
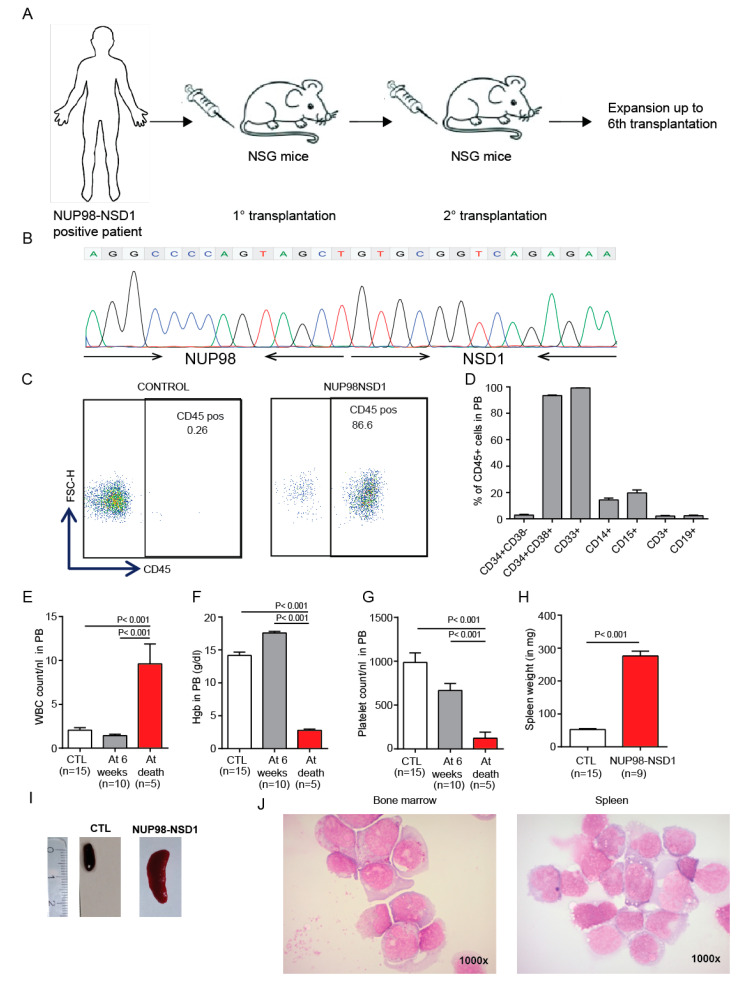
Establishment and characterization of a NUP98-NSD1 patient-derived xenograft (PDX) model. (**A**) Development of a patient-derived xenograft (PDX) model from a NUP98-NSD1 fusion-positive patient. (**B**) Identification of the NUP98-NSD1 fusion through Sanger sequencing, where the 5′end (exon 11) of NUP98 is fused to the 3′end (exon6) of NSD1. (**C**) Engraftment of human CD45-positive NUP98-NSD1 AML cells in peripheral blood 10 weeks after transplantation. (**D**) Immunophenotype of human CD45-positive NUP98-NSD1 AML cells in the peripheral blood of PDX mice. (**E**) WBC count in peripheral blood of PDX-AML mice at 6 weeks after transplantation (before the development of AML) and at sacrifice (6th transplantation) (mean ± SEM). (**F**) Hemoglobin of PDX-AML mice at 6 weeks after transplantation (before the development of AML) and at sacrifice (6th transplantation) (mean ± SEM). (**G**) Platelet count in peripheral blood of PDX-AML mice at 6 weeks after transplantation (before the development of AML) and at sacrifice (6th transplantation) (mean ± SEM). (**H**) Spleen weight at death of the PDX model (6th transplantation) (mean ± SEM). (**I**) Representative image of a spleen at sacrifice/death. (**J**) Representative Wright–Giemsa-stained cytospin preparations of bone marrow, spleen, and blood cells from moribund mice (1000×) (6th transplantation).

To establish a NUP98-NSD1 PDX model, we transplanted bone marrow cells from three NUP98-NSD1-positive AML patients and serially transplanted the cells from one model that showed engraftment (Figure 4A). The NUP98-NSD1 fusion in this model consisted of a fusion between exon 11 of NUP98 and exon 5 of NSD1. This NUP98-NSD1-positive PDX model was analyzed in the 6th transplantation and showed AML with normal karyotype, while it was wildtype for genes frequently mutated in leukemia, such as *FLT3, NRAS, NPM1, IDH1/2*, and *DNMT3A* (Table S1). Interestingly, we found that the variant allele frequency of mutations in *BCOR* and *PTPN11* were consistent throughout all transplantations. However, the variant allele frequency of mutated CEBPA decreased, while that of mutated *WT1* increased in successive transplantations. This PDX model may explain why *WT1* mutations often co-occur with the NUP98-NSD1 fusion in AML patients (Figure S7A–D). These patient cells have been serially transplanted up to the 6th generation and the presence of the NUP98-NSD1 fusion gene was confirmed through all transplantations, suggesting that the fusion gene is required for leukemia development and maintenance. In later transplantations, a minor clone of NUP98-NSD1 was identified, which had an extra GCT in the fusion junction. In our PDX model, the engraftment of human leukemia cells reached up to 86% after ten weeks of transplantation (Figure 4C) and the mice became moribund after 11–14 weeks. Immunophenotypic analysis of human CD45+ cells showed high expression of myeloid markers (CD33+) and low expression of lymphoid markers (CD3+, CD19+) confirming a diagnosis of AML (Figure 4D). Additionally, it showed higher expression of hematopoietic progenitor markers CD34+CD38+, indicating a more immature leukemic population. Between 6 weeks after transplantation and time of sacrifice, WBC counts increased, while hemoglobin and platelet counts decreased (Figure 4E–G), and mice presented with large spleens at time of sacrifice (Figure 4H,J). Cytospin analysis of bone marrow and spleen cells confirmed the diagnosis of AML by a high blast count (Figure 4J). In summary, our serially transplantable PDX model is a highly clinically relevant model and provides a platform to test different therapeutics for their in vivo efficacy.

### 3.5. Treatment of the NUP98-NSD1 PDX Model with NUP98-NSD1 siRNA-LNPs Prolongs Survival of Mice

**Figure 5:**
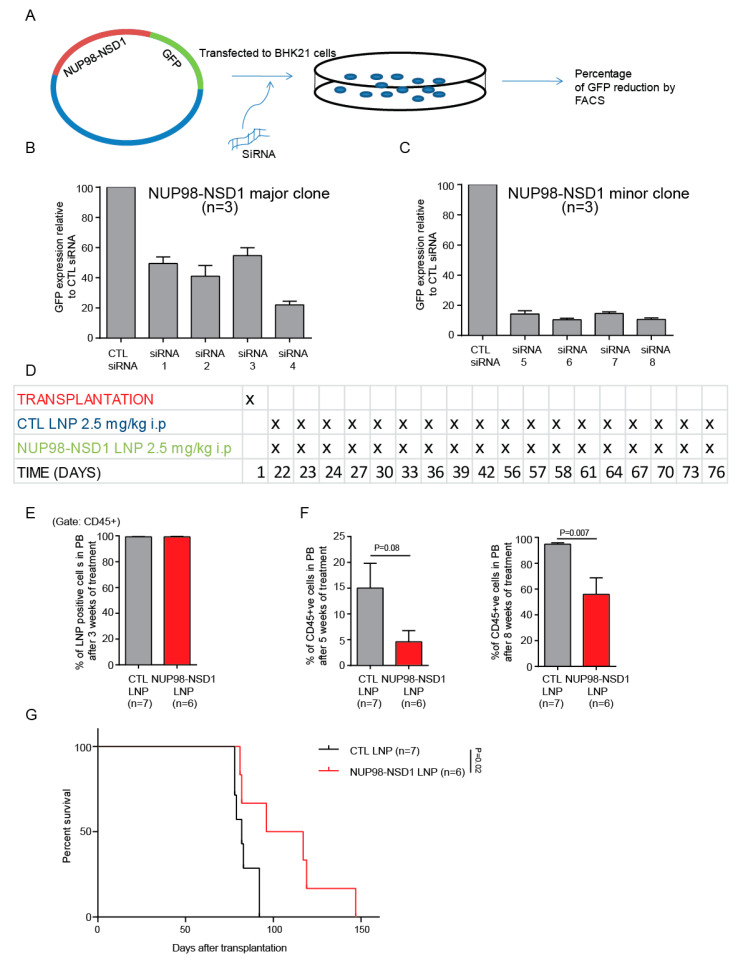
Treatment of the NUP98-NSD1 PDX model with NUP98-NSD1 siRNA-LNPs prolongs survival of mice. (**A**) Schematic presentation of the siRNA screening procedure. BHK21 cells were transfected with siRNAs and plasmid. Three days post-transfection, GFP expression was measured by flow cytometry. (**B**) Efficacy of four siRNAs against the major clone of NUP98-NSD1 (mean ± SEM). The CTL siRNA was directed against BCR-ABL, which is not expressed in the studied cells. (**C**) Efficacy of 4 siRNAs against the minor clone of NUP98-NSD1 (mean ± SEM). The CTL siRNA was directed against BCR-ABL, which is not expressed in the studied cells. (**D**) Schematic outline of the LNP-siRNA treatment schedule in the NUP98-NSD1 PDX model. The LNP-siRNA treatment was started 3 weeks after transplantation of NUP98-NSD1 PDX cells and was applied in 2 cycles of 3 weeks each. Control and NUP98-NSD1 siRNA formulations were injected ad day 1, 2, and 3 and then every 3rd day in each cycle with a dose of 2.5 mg/kg. (**E**) LNP uptake in CD45+ AML cells in peripheral blood of treated mice after 3 weeks of treatment. (**F**) Engraftment of human CD45-positive AML cells at 8 and 11 weeks of transplantation (after 5 and 8 weeks of treatment) in peripheral blood of NUP98-NSD1 AML PDX mice treated with control or NUP98-NSD1 siRNA/LNP formulations (mean ± SEM). (**G**) Survival of NUP98-NSD1 AML-PDX mice treated with LNP-siRNA (CTRL or NUP98-NSD1).

To establish a novel treatment for NUP98-NSD1-positive AML, we aimed to directly inhibit the fusion gene by siRNA. We designed four different siRNAs, which span the translocation junction for both the major and minor clones of NUP98-NSD1. We tested the efficacy of each siRNA and identified the most potent siRNA using our siRNA screening system (Figure 5A). siRNA4 and siRNA6 showed the maximum knockdown for the major and minor clones, respectively (Figure 5B,C). We chemically modified the effective siRNAs to increase their in vivo stability and re-confirmed their continued activity against their targets (Figure S8A,B). The siRNAs (major clone: minor clone in 3:1 ratio) were packaged into LNPs using the NanoAssemblr (95% packaging efficacy). The average size of the LNPs was 45–65 nm (Figure S9A,B) and the average zeta potential was −1.5 mv to −4 mv. As our transduced NUP98-NSD1 cell line model is driven by the MSCV promoter leading to strong constitutive expression of the fusion oncogene, we did not evaluate siRNA knockdown efficacy in these cells. Instead, we tested the efficacy of our siRNA/LNP formulations in vitro using NUP98-NSD1 PDX cells, which express the fusion gene from an endogenous promoter. We demonstrated a significant increase in apoptosis and a significant reduction in the mRNA level of the NUP98-NSD1 fusion gene after NUP98-NSD1-LNP treatment compared to CTL-LNP treatment (Figure S10A,B). LNPs were injected intraperitoneally on days 1, 2, and 3 and then every third day for 3 weeks starting 3 weeks after transplantation. This treatment was repeated for a second 3-week cycle starting 8 weeks after transplantation (Figure 5D and Figure S11A). We randomized the mice before the start of the treatment in order to have similar engraftment levels in both the CTL-LNP and NUP98-NSD1-LNP treatment group (Figure S11B). After 3 weeks of treatment, LNP uptake into human CD45+ cells in peripheral blood was 99.3% and 99.2% in the CTL-LNP and NUP98-NSD1-LNP groups, respectively (Figure 5E). The mean engraftment was significantly lower in NUP98-NSD1-LNP compared to CTL-LNP treated mice 5 and 8 weeks after the start of the treatment (Figure 5F). Importantly, the NUP98-NSD1 siRNA-LNP treated mice showed a significant survival benefit compared to CTL siRNA-LNP-treated mice (median survival 106 vs. 82 days after transplantation, *p* = 0.02; Figure 5G). No significant difference in blood counts was observed during different time points after the treatment (Figure S11C–E). At the time of sacrifice, we did not find any significant difference in engraftment in the bone marrow and spleen of CTL-LNP and NUP98-NSD1-LNP groups (Figure S11F,G). In summary, we show that targeting the NUP98-NSD1 fusion oncogene can decrease the leukemic burden and significantly improves the survival of NUP98-NSD1-positive mice.

## 4. Discussion

We studied the oncogenic activity of the NUP98-NSD1 fusion and the frequently co-occurring mutation NRASG12D in vitro and in vivo. We showed that NUP98-NSD1 but not mutated *NRAS* has leukemogenic activity in vitro and in vivo, while mutated *NRAS* strongly enhances the leukemogenic potency of NUP98-NSD1, supporting the frequent co-occurrence of these mutations. Mechanistically, NUP98-NSD1 activates the distal *Hoxa* cluster genes, while mutated *NRAS* dysregulates different pathways involving inflammation, transport, migration, metabolism, and angiogenesis. In a human AML PDX model of NUP98-NSD1, we show that inhibition of the fusion by a novel LNP/siRNA formulation has ant leukemic activity and prolongs the survival of mice.

It has previously been shown that overexpression of the NUP98-NSD1 fusion alone induces an AML-like disease in mice [15], while, similar to our finding that NRASG12D enhances the leukemogenicity of NUP98-NSD1, overexpression of the NUP98-NSD1 fusion in association with the FLT3-ITD mutation causes leukemia [32]. Here, we show that constitutive expression of the NUP98-NSD1 fusion alone induces leukemia with long latency, and that complementation with NRASG12D accelerates the disease to an aggressive leukemia. A higher proliferation index, inhibition of apoptosis, higher colony forming potential in vitro, and leukemogenic potential in vivo of NUP98-NSD1 compared to NRASG12D suggest that NUP98-NSD1 is the oncogenic driver in this leukemia. This is supported by the work demonstrating that NUP98-HOXD13 expression in hematopoiesis induces leukemia with long latency, suggesting that additional cooperative genetic events like mutated *NRAS, KRAS,* and *FLT3* reduce the disease latency [33,34,35]. In addition, we developed an additional NUP98-NSD1 PDX model [36], which engrafted up to the 6th generation. We observed an increase in variant allele frequency of *WT1* from the first to the sixth transplantation that exemplifies the functional cooperation of NUP98-NSD1 and *WT1* in AML progression, suggesting that our PDX model represents the human disease well and provides a suitable platform to develop potential therapeutics.

Besides the NUP98-NSD1 fusion, other fusion partners of *NUP98* that co-occur with mutated *NRAS* have been reported [4]. RUNX1-RUNX1T1, CBFB-MYH11, AML1-ETO, and MLL rearranged leukemias also co-occur and synergize with *NRAS* mutations [37,38,39,40]. High expression of CD11b in the NUP98-NSD1 syngeneic model and high expression of CD33 in the PDX model indicate that the NUP98-NSD1 fusion skews leukemic development towards the myeloid lineage in both murine and PDX models, which differs from other *NUP98* fusions such as NUP98-HOXD13 that differentiate into a wide spectrum of hematological malignancies such as erythroid, myeloid, and T cell leukemia [41]. We observed that overexpression of NUP98-NSD1 induced genes of the *Hoxa* cluster in the murine model, which is consistent with earlier findings [15]. Dysregulation of the *Hox* pathway has also been reported for other fusion partners of *NUP98* such as *HOXA9*, *HOXD13*, *PHF23*, and *TOP1* [42], while NUP98 alone has been implicated in aberrant H3K4 trimethylation at the Hox locus in hematopoietic cells [43]. Apart from the NUP98 fusions, *Hox* gene dysregulation has also been associated with MLL fusions, the CALM-AF10 fusion, and *NPM1* and *IDH1* mutations in leukemia [44,45,46,47]. Additionally, our gene expression data implicate increased MAPK13, cystatin family genes, and cyclin D1 as important downstream pathways that mediate the effects of NRASG12D on the NUP98-NSD1 leukemic model.

We built on our experience in targeting fusion genes and associated transcription factors to inhibit the dominant driver in the NUP98-NSD1 PDX model by siRNA/LNPs. Targeted inhibition of NUP98-NSD1 using a siRNA-LNP treatment showed a survival benefit compared to control LNPs. Previous work by our group also showed that inhibition of the driver fusion genes using siRNA-LNP treatment reduces the leukemic burden in Chronic myelogenous leukemia (CML) and Acute lymphoblastic leukemia(ALL) models [48,49]. Until August 2019, over 25 nanoparticle treatments have been approved by the Food and Drug Administration (FDA) or European Medicines Agency (EMA) for clinical use, which underscores the translational potential of our study for the treatment of NUP98-NSD1-positive AML patients [50]. Ligand conjugated delivery of LNPs can be used to specifically target leukemic stem cells over normal hematopoietic stem cells without disrupting normal hematopoiesis. Different receptors like CD123, CD33, or CLL1 were found to be expressed in leukemia stem cells, but they are also expressed on normal stem cells and their expression varies across different subtypes of leukemia [51]. However, since these fusions appear only in leukemic cells, fusion driven leukemias can be directly targeted by an untargeted delivery of LNP/siRNAs with limited toxicity. Therefore, our treatment approach can be utilized for treatment of a broad range of leukemias that are associated with fusion genes.

In summary, we developed and characterized transplantable AML models of NUP98-NSD1 fusion and validated the synergistic effect of NUP98-NSD1 and NRASG12D. Additionally, a novel NUP98-NSD1 siRNA-LNP formulation was validated as an effective treatment strategy for NUP98-NSD1-positive AML, supporting the preclinical and clinical development of this treatment approach to improve the outcome of NUP98-NSD1-positive AML patients.

## 5. Conclusions

Our study validated the efficacy of a targeted siRNA-LNP formulation for NUP98-NSD1 AML that supports further development and evaluation of this approach for other chemoresistant AML subgroups.

## Supplementary Materials

The following are available online at https://www.mdpi.com/2072-6694/12/10/2766/s1, Figure S1: Transduction of mouse bone marrow cells with NUP98-NSD1, but not NRASG12D, induces myeloid transformation, Figure S2: NUP98-NSD1 and NUP98-NSD1+NRASG12D induce myeloid leukemia in vivo, Figure S3: Immunophenotypic characterization of moribund mice which received transplants of NUP98-NSD1+NRASG12D transduced cells, Figure S4: Secondary transplantation of NUP98-NSD1 and NUP98-NSD1+ NRASG12D leads to an aggressive AML, Figure S5: Gene expression analysis of RNAseq data from in vitro expanded NRASG12D, NUP98-NSD1 and NUP98-NSD1+NRASG12D transduced mouse bone marrow cells, Figure S6: Gene set enrichment analysis of NUP98-NSD1 transduced mouse bone marrow cells (NRASG12D vs NUP98-NSD1) and gene set enrichment analysis of NRASG12D transduced mouse bone marrow cells (NUP98-NSD1+NRASG12D vs NUP98-NSD1), Figure S7: Identification and variant allele frequency of mutations in the NUP98-NSD1 PDX model from first to sixth recipient, Figure S8: Identification and validation of siRNAs against the NUP98-NSD1 fusions, Figure S9: Characterization of the siRNA-LNP formulations using the zetasizer, Figure S10: Efficacy of siRNA-LNP formulations in ex vivo PDX cells, Figure S11: Engraftment of leukemic cells and blood counts at different time points during LNP treatment, Table S1: Mutational status of NUP98-NSD1 6th PDX bone marrow cells, Table S2: List of primers used for quantitative RT-PCR, Table S3: List of siRNA sequences.
