# Supplementary material for: Targeted Inhibition of the NUP98-NSD1 Fusion Oncogene in Acute Myeloid Leukemia
Source: Cancers (Basel). 2020 Sep 26;12(10):2766. doi: 10.3390/cancers12102766 (PMC7600396; doi:10.3390/cancers12102766)
Supplement: Supplementary file 1 [file cancers-12-02766-s001.zip › 200925_Mohanty eta al. Supplementary data.docx]

**Targeted Inhibition of the NUP98-NSD1 Fusion Oncogene in Acute Myeloid Leukemia**

Sagarajit Mohanty^1^, Nidhi Jyotsana^2^ Amit Sharma^1^, Arnold Kloos^1^, Razif Gabdoulline^1^, Basem Othman^1^, Courteney K. Lai1, Renate Schottmann^1^, Madhvi Mandhania^3^, Johannes Schmoellerl^4^, Florian Grebien^4^, Euan Ramsay^5^, Anitha Thomas^5^, Hans-Peter Vornlocher^6^, Arnold Ganser^1^, Felicitas Thol^1^, Michael Heuser^1^

## Supplemental Data

**Supplemental Methods**

**Mouse transplantation and treatment**

For the syngeneic mouse model, 1 x 10^6^ transformed murine cells accompanied by 1 x 10^5^ freshly isolated BM cells from helper mice were intravenously injected into irradiated female C57BL6/J mice (8 Gy single dose total body irradiation). For the patient derived xenograft (PDX) model, 1 x 10^6^ cells from an AML patient were intraveneously injected into irradiated female NOD.Cg-*Prkdc^scid^ Il2rg^tm1Wjl^*/SzJ(NSG) mice (2.5 Gy). For serial transplantation, bone marrow or spleen cells from a moribund mouse were transplanted into syngeneic mice. Engraftment was monitored through flow cytometric analysis of fluorescence marker expression in peripheral blood (PB) cells obtained through cheek or retro-orbital vein bleeds. Engrafted cells of human origin in NSG mice were monitored through expression of human CD45 positive peripheral blood (PB) cells using a flow cytometer. Blood counts (WBC, hemoglobin and platelets) were obtained using an ABC Vet Automated Blood counter (Scil animal care company GmbH, Viernheim, Germany). Engraftment and expression of differentiation markers were analyzed in peripheral blood, spleen and bone marrow of mice at time of sacrifice (for details see below). The treatment schedule and the dose for the treatment of PDX mice the siRNA-LNP formulation is shown in the results section.

For in vitro LNP treatment, NUP98-NSD1-PDX cells were isolated from mouse bone marrow by immunomagnetic selection (Mouse Cell Depletion Kit, Miltenyi Biotec, Bergisch Gladbach, Germany). 1x10^5^ PDX cells were cultured in a 24 well plate and pre-stimulated in Iscove’s modified Dulbecco’s medium (IMDM, StemCell Technologies, Cologne, Germany) supplemented with 20% bovine serum albumin, insulin and transferrin (BIT 9500, StemCell Technologies, Cologne, Germany), 0.1mM 2-mercaptoethanol (Sigma-Aldrich, Munich, Germany), 2 mM L-Glutamine (Gibco, Thermofisher Scientific, Bremen, Germany), 20 ng/ml human IL-6, 20 ng/ml human IL-3, 20 ng/ml human granulocyte colony-stimulating factor (G-CSF), 100 ng/ml human stem cell factor (SCF), 50 ng/ml thrombopoietin (TPO) and 100 ng/ml FLT3-ligand (all from PeproTech, Hamburg, Germany) at 37°C with 5% CO_2_. PDX cells were treated on day 0 and day 2 at a dose of 1 μg/ml and cells were collected on day 4 for apoptosis assays and RT-PCR.

**Clonogenic progenitor assay**

Colony-forming cell (CFC) units were assayed in methylcellulose (Methocult M3234; StemCell Technologies, Inc., Vancouver, BC, Canada) supplemented with 10 ng/mL murine IL3, 10 ng/mL human IL6, 50 ng/mL SCF, and 3 U/mL EPO (PeproTech, Hamburg, Germany) for mouse cells. One thousand sorted NRAS BFP+, NUP98-NSD1 GFP+ and NUP98-NSD1+NRAS GFP+BFP+ cells were plated in duplicate and repeated with 3 independent clones. After one week of seeding, colonies were counted under the Olympus CKX31 (Olympus, Tokyo, Japan) microscope. For serial plating, the same numbers of cells as for the first plating were plated.

**Morphologic analysis**

Cytospins were prepared from cells from mouse bone marrow, spleen and peripheral blood and stained with Wright-Giemsa stain. Morphology was analysed via Zeiss Axioscope A1 using the Axiocamera 5s microscope with Zeiss immersol medium, and images were taken with 1000x magnification and processed with the Zen 2.6 lite (blue) software (Zeiss, Jena, Germany).

**Antibodies for immunophenotyping**

Antibodies used for immunophenotyping of murine cells were Gr-1-PE (clone-RB6-8C5), B220-PE (RA3-6B2), Sca-1-PE (D7), CD4-PE (H129.19), CD8-APC(53-6.7) and c-kit-APC (2B8) from BD Biosciences, and CD11b-APC (M 1/70), F4/80(BMB) and IgER (MAR-1) from eBioscience. For immunophenotyping human cells, CD45-FITC (HI30,CAT 555482), CD45-PE (H130, CAT 555483), CD45-APC (HI30,CAT 555485), CD33-PE (WM53, CAT 555450), CD38-PE (HB-7, CAT 345806), CD34-APC (8G12, CAT 345804), CD14-APC (M5E2 , CAT 555399), CD3-PE (UCHT1, CAT 555333) and CD19-APC (HIB19, CAT 555415) from BD Biosciences and CD15-PE (80H5, CAT IM1954U) from Beckman Coulter ([Krefeld](https://en.wikipedia.org/wiki/Krefeld), Germany) were used. Flow cytometry data were analyzed using the FlowJo software (V10.0.7, TreeStar, Ashland, OR, USA).

**siRNA screening**

1x 105 BHK-21 cells were seeded in 1 mL medium (DMEM+10%FCS+1%P/S) in a 24-well plate. After 12 hours, the screening plasmid (MSCV-NUP98-NSD1-IRES-GFP), control LNSN plasmid (low-affinity nerve growth factor receptor (LNGFR) expressing plasmid)([1](#_ENREF_1)) and siRNA were co-transfected. The transfection was facilitated by lipofectamine 3000 (Thermo Fisher Scientific GmbH, Bremen, Germany, CAT L3000015). Seventy-two hours post-transfection, cells were stained with phycoerythrin (PE) mouse antihuman CD271 antibody (BD PharMingen, San Diego, CA, CAT 557196) for 15 minutes at room temperature and EGFP and LNGFR expression were assessed using the BD LSRII flow cytometer. As both fusion genes and GFP are expressed from the same promotor, the reduction of GFP expression was used to quantify the knockdown of the target gene. All siRNA sequences are provided in Supplementary Table S3.

**Lipid nanoparticle (LNPs)/siRNA formulation**

The lipid mixture used to package our target siRNAs contained a proprietary lipid mix (SUB9KIT, Precision Nanosystems, Vancouver, Canada, with minor modifications) consisting of an ionizable amino lipid, a phosphatidylcholine (1, 2-distearoyl-sn-glycero-3-phosphocholine, DSPC), cholesterol and a coat lipid (polyethylene glycol-dimyristoylglycerol, PEG-DMG) at a molar ratio of 50:10:38.5:1.5. These ionizable cationic LNPs have pKa values below 7 which permit siRNA encapsulation at low pH and a relatively neutral surface at physiological pH in order to maximize cellular uptake. Our LNPs also include DiI labelled lipids, which has an excitation wavelength of 549 nm and emission wavelength of 565 nm, which allowed measurement of LNP uptake by flow cytometry. Our LNP formulation was made with 1.5% PEG-DMG to increase stability and circulation half-life *in vivo*. LNP-siRNA formulations were formed by injecting the lipid/ethanol solution into the first inlet, and a low pH aqueous buffer (sodium acetate buffer 25mM, pH 4) containing the siRNA in the second inlet of the microfluidic chip of the NanoAssemblr (Precision Nanosystems, Vancouver, BC). After microfluidic mixing at a flow rate of 12 mL/min, with siRNA to lipid flow ratio of 3:1, the encapsulated LNPs underwent dialysis overnight at 4°C and were filtered using a 0.22 micron syringe filter before use.

**Quantification of encapsulation efficiency of LNPs**

The Ribogreen assay was used to quantify the siRNA encapsulated within the LNPs. Encapsulation efficiency of LNPs was determined by measuring the fluorescence upon the addition of RiboGreen (Molecular Probes, Eugene, OR) to the unencapsulated siRNA-LNP (Fi) and comparing this value to the total siRNA content obtained upon lysis of the LNP by 1% Triton X-100 (Ft). Percent encapsulation was calculated by the formula (Ft – Fi)/Ft x 100.

**Measurement of size and Zeta potential of LNPs**

The Zetasizer ZS instrument (Malvern Instruments Ltd, Worcestershire, United Kingdom) was used to determine the size and zeta potential of the LNPs. LNPs were diluted with PBS to get a good scattering and the measurement was performed at 25 °C.

## Supplemental Tables

**Supplementary Table S1:** Mutational status of NUP98-NSD1 6^th^ PDX bone marrow cells

| Gene name | Mutation status | Gene name | Mutation status |
| --- | --- | --- | --- |
| ASXL1 | WT | MPL | WT |
| ASXL2 | WT | MYC | WT |
| BCOR | mut | NF1 | WT |
| BCORL1 | WT | NPM1 | WT |
| BRAF | WT | NRAS | WT |
| CALR | WT | PHF6 | WT |
| CBL | WT | PPM1D | WT |
| CEBPA | WT | PTPN11 | mut |
| CSF3R | WT | RAD21 | WT |
| CSNK1A1 | WT | RUNX1 | WT |
| DDX41 | WT | SETBP1 | WT |
| DNMT3A | WT | SF3B1 | WT |
| ETNK1 | WT | SMC1A | WT |
| ETV6 | WT | SMC3 | WT |
| EZH2 | WT | SRSF2 | WT |
| FLT3 | WT | STAG1 | WT |
| GATA2 | WT | STAG2 | WT |
| IDH1 | WT | TET2 | WT |
| IDH2 | WT | TP53 | WT |
| JAK2 | WT | U2AF1 | WT |
| KDM6A | WT | WT1 | mut |
| KIT | WT | ZBTB7A | WT |
| KRAS | WT | ZRSR2 | WT |

Abbreviation: WT, wildtype; mut, mutation.

BCOR mut (NM_001123385.1:c.3239-2A>T)

PTPN11 mut (NM_002834.3:c.1504T>C,p.Ser502Pro)

WT1 mut (NM_024426.4:c.1385G>A,p.Arg462Gln)

CEBPA mut (NM_004364.3:c.308_313delGCGGCG,p.Gly103_Gly104del)

**Supplementary Table S2:** **List of primers used for quantitative RT-PCR.**

| Primer | Sequence |
| --- | --- |
| Abl_F | TGGGTAGTGGAGTGTGGTGATGAG |
| Abl_R | AACACTGCCTCTGATGGCAAGC |
| Hoxa7_F | AGTTCAGGACCCGACAGGA |
| Hoxa7_R | CGTCAGGTAGCGGTTGAAAT |
| Hoxa9_F | CCCATCGATCCCAATAACC |
| Hoxa9_R | CTTCTCCAGTTCCAGCGTCT |
| Hoxa10_F | GACTCCCTGGGCAGTTCC |
| Hoxa10_R | TGTAAGGGCAGCGTTTCTTC |

**Supplementary Table S3:** **List of siRNA sequences.**

All siRNAs were purchased either Dharmacon (Freiburg, Germany) or Axolabs GmbH (Kulmbach, Germany). NUP98-NSD1 siRNA 1, 2, 3, 4, 5, 6, 7, 8 and NUP98-NSD1 siRNA6 mod were purchased from Dharmacon (Freiburg, Germany) and NUP98-NSD1 siRNA4 mod1 and mod2, CTRL BCR-ABL siRNA and CTRL BCR-ABL siRNAmod were purchased from Axolabs GmbH (Kulmbach, Germany).

| siRNA | Sequence |
| --- | --- |
| NUP98-NSD1 siRNA 1 sense | 5´GCCCCAGUAGCUGUGCGGUdTdT 3‘ |
| NUP98-NSD1 siRNA 1 antisense | 5´ACCGCACAGCUACUGGGGCdTdT 3‘ |
| NUP98-NSD1 siRNA 2 sense | 5´CCCCAGUAGCUGUGCGGUCdTdT 3‘ |
| NUP98-NSD1 siRNA 2 antisense | 5´GACCGCACAGCUACUGGGGdTdT 3‘ |
| NUP98-NSD1 siRNA 3 sense | 5´GGCCCCAGUAGCUGUGCGGdTdT 3‘ |
| NUP98-NSD1 siRNA 3 antisense | 5´CCGCACAGCUACUGGGGCCdTdT 3‘ |
| NUP98-NSD1 siRNA 4 sense | 5´CCCAGUAGCUGUGCGGUCAdTdT 3‘ |
| NUP98-NSD1 siRNA 4 antisense | 5´UGACCGCACAGCUACUGGGdTdT 3‘ |
| NUP98-NSD1 siRNA 5 sense | 5' AGUAGCUGCUGUGCGGUCAdTdT 3' |
| NUP98-NSD1 siRNA 5 antisense | 5' UGACCGCACAGCAGCUACUdTdT 3' |
| NUP98-NSD1 siRNA 6 sense | 5' CAGUAGCUGCUGUGCGGUCdTdT 3' |
| NUP98-NSD1 siRNA 6 antisense | 5' GACCGCACAGCAGCUACUGdTdT 3' |
| NUP98-NSD1 siRNA 7 sense | 5' GUAGCUGCUGUGCGGUCAGdTdT 3' |
| NUP98-NSD1 siRNA 7 antisense | 5' CUGACCGCACAGCAGCUACdTdT 3' |
| NUP98-NSD1 siRNA 8 sense | 5' UAGCUGCUGUGCGGUCAGAdTdT 3' |
| NUP98-NSD1 siRNA 8 antisense | 5' UCUGACCGCACAGCAGCUAdTdT 3' |
| NUP98-NSD1 siRNA 4 sense mod1 | 5' cccAGuAGcuGuGcGGucAdTdT3' |
| NUP98-NSD1 siRNA 4 antisense mod1 | 5' UGACCGcAcAGCuACUGGGdTdT3' |
| NUP98-NSD1 siRNA 4 sense mod2 | 5'ccCfaGfuAfgCfuGfuGfcGfgUfcAfdTdT3' |
| NUP98-NSD1 siRNA 4 antisense mod2 | 5'UfGfaCfcGfcAfcAfgCfuAfcUfgGfgdTdT3' |
| NUP98-NSD1 siRNA 6 sense mod | On Target Plus modification from Dharmacon |
| NUP98-NSD1 siRNA 6 antisense mod | On Target Plus modification from Dharmacon |
| CTRL BCR-ABL siRNA sense | 5' GCAGAGUUCAAAAGCCCUUdTdT 3' |
| CTRL BCR-ABL siRNA anti sense | 5' AAGGGCUUUUGAACUCUGCdTdT 3' |
| CTRL BCR-ABL siRNA sense mod | 5’ gcAfGAfgUfuCfaAfaAfgCfcCfuUfdTsdT 3’ |
| CTRL BCR-ABL siRNA anti sense mod | 5’ AfAfgGfgCfuUfuUfgAfaCfuCfuGfcdTsdT 3’ |

Abbreviations: mod, modification; CTRL, control.

Note: a, c, g, u: 2'-O-methyl residues; Af, Cf, Gf, Uf: 2'-F residues; dT: desoxy-T; s: phosphorothioate.

## Supplemental Figures


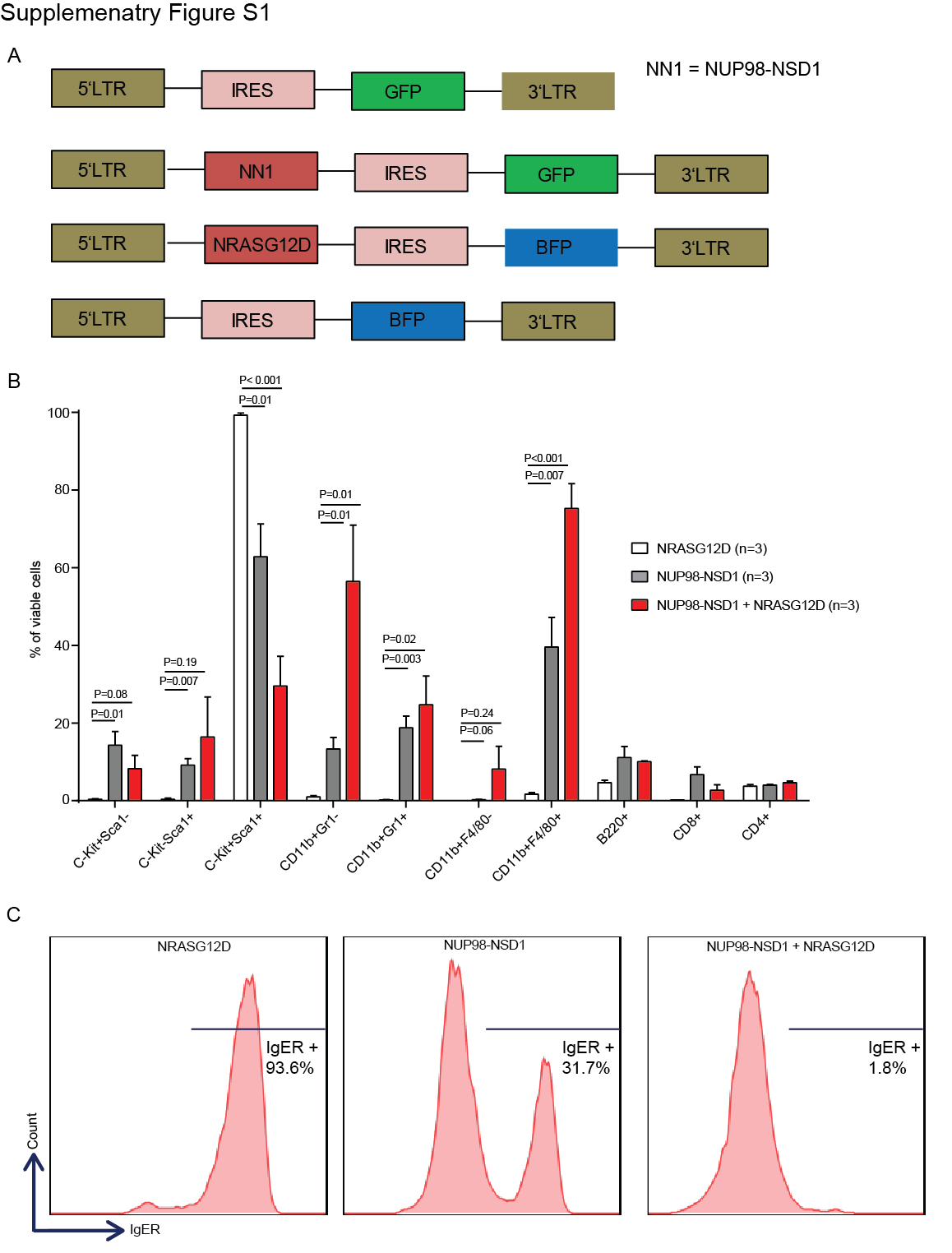


**Supplementary Figure S1.** **Transduction of mouse bone marrow cells with NUP98-NSD1, but not NRASG12D, induces myeloid transformation.**

1. Schematic of the MSCV-based retroviral constructs. LTR5′, 5′ long terminal repeats; LTR3′, 3′ long terminal repeats; IRES, internal ribosome entry site.
2. Immunophenotype of cells transduced with the indicated constructs (mean ± SEM).
3. Representative flow cytometric histogram shows expression of IgER in mouse bone marrow cells transduced with the indicated constructs.


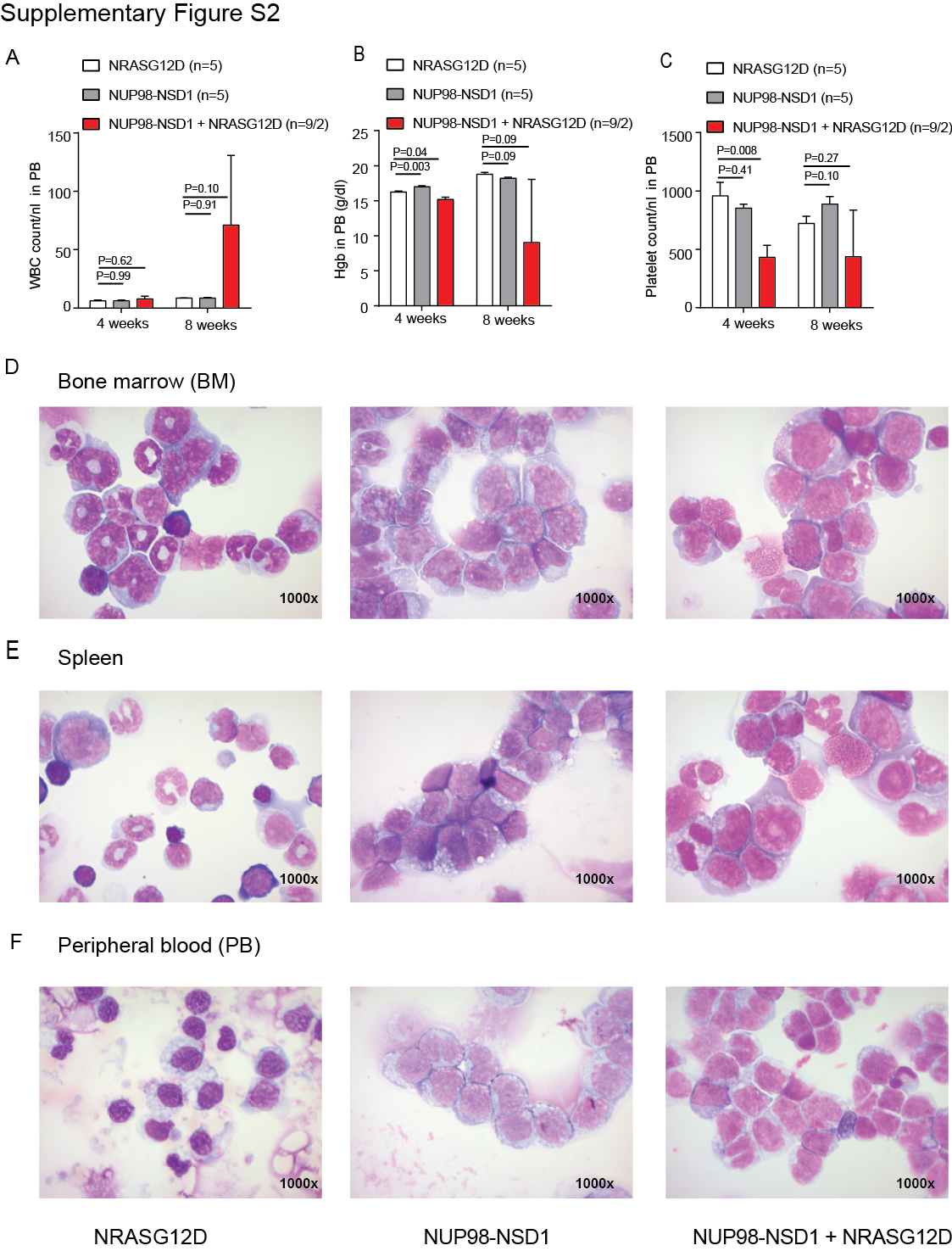


**Supplementary Figure S2.** **NUP98-NSD1 and NUP98-NSD1+NRASG12D induce myeloid leukemia *in vivo*.**

1. White blood cell count in peripheral blood of mice transplanted with NRASG12D, NUP98-NSD1 and NUP98-NSD1+NRASG12D transduced bone marrow cells (mean ± SEM).
2. Hemoglobin levels in peripheral blood of mice transplanted with NRASG12D, NUP98-NSD1 and NUP98-NSD1+NRASG12D transduced bone marrow cells (mean ± SEM).
3. Platelet count in peripheral blood transplanted with NRASG12D, NUP98-NSD1 and NUP98-NSD1+NRASG12D transduced bone marrow cells (mean ± SEM).
4. Representative Wright-Giemsa–stained cytospin preparations from bone marrow cells of moribund mice (1000x).
5. Representative Wright-Giemsa–stained cytospin preparations from spleen cells of moribund mice (1000x).
6. Representative Wright-Giemsa–stained cytospin preparations from blood cells of moribund mice (1000x).


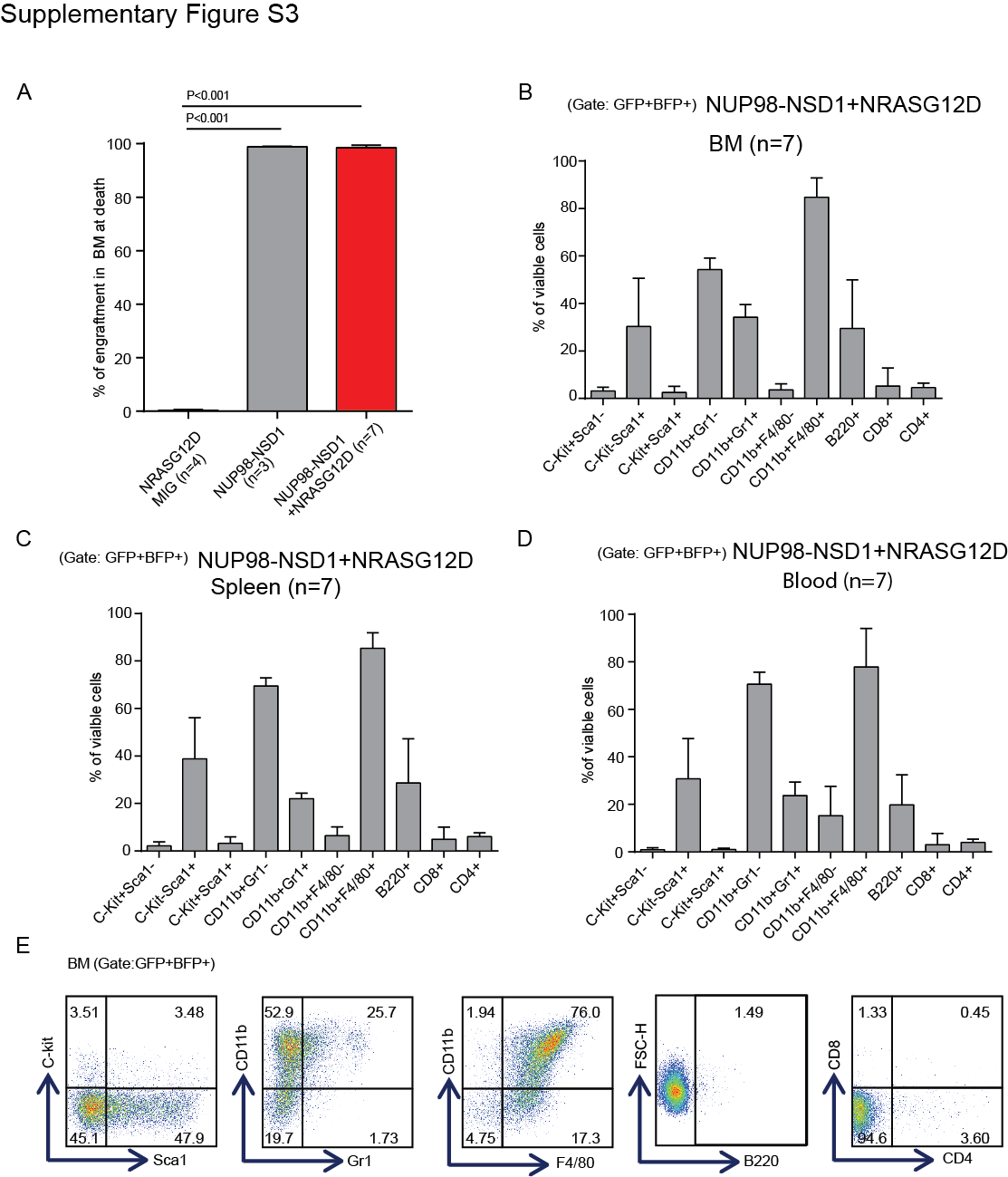


**Supplementary Figure S3.** **Immunophenotypic characterization of moribund mice which received transplants of NUP98-NSD1+NRASG12D transduced cells.**

1. Engraftment in bone marrow at death of mice which received transplants of the indicated cells.
2. Immunophenotype of bone marrow cells from moribund NUP98-NSD1+NRASG12D mice (mean ± SEM).
3. Immunophenotype of spleen cells from moribund NUP98-NSD1+NRASG12D mice (mean ± SEM).
4. Immunophenotype of blood cells from moribund NUP98-NSD1+NRASG12D mice (mean ± SEM).
5. Representative immunophenotype of bone marrow cells from moribund NUP98-NSD1+NRASG12D mice.


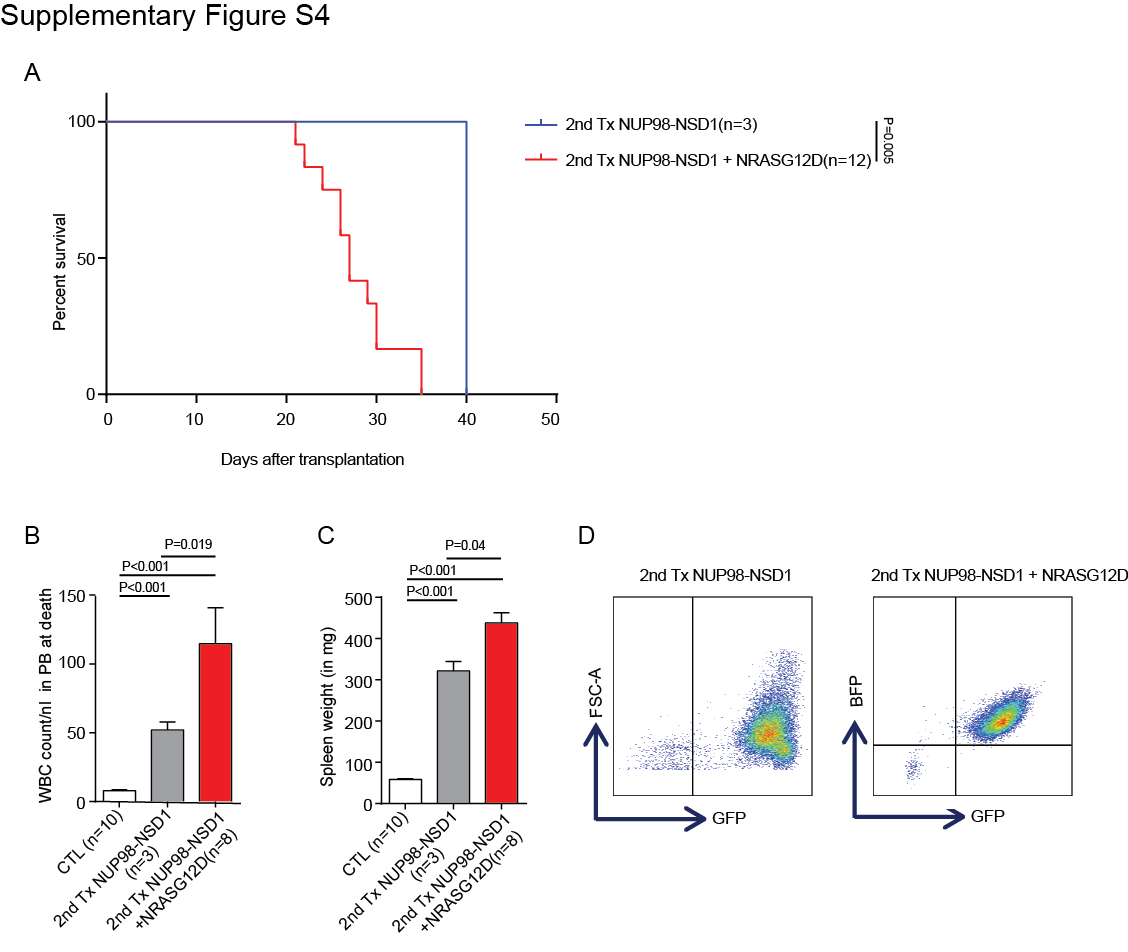


**Supplementary Figure S4.** **Secondary transplantation of NUP98-NSD1 and NUP98-NSD1+ NRASG12D leads to an aggressive AML.**

1. Survival curve of mice that received transplants of bone marrow cells from primary transplantations.
2. Peripheral blood white blood cell count at time of sacrifice of transduced cells from primary transplantation (mean ± SEM). Data from mice transplanted with Hoxa9-MIGR1 transduced cells (CTL) and WBC count was obtained 6 weeks after transplantation.
3. Average spleen weight of mice from primary transplantation at time of sacrifice (mean ± SEM). Spleen weights from mice transplanted with Hoxa9-MIGR1 transduced cells (CTL) were measured 3 months after transplantation, at the time of sacrifice.
4. Representative flow cytometric analysis shows expression of transduced cells in BM of the mice at death.


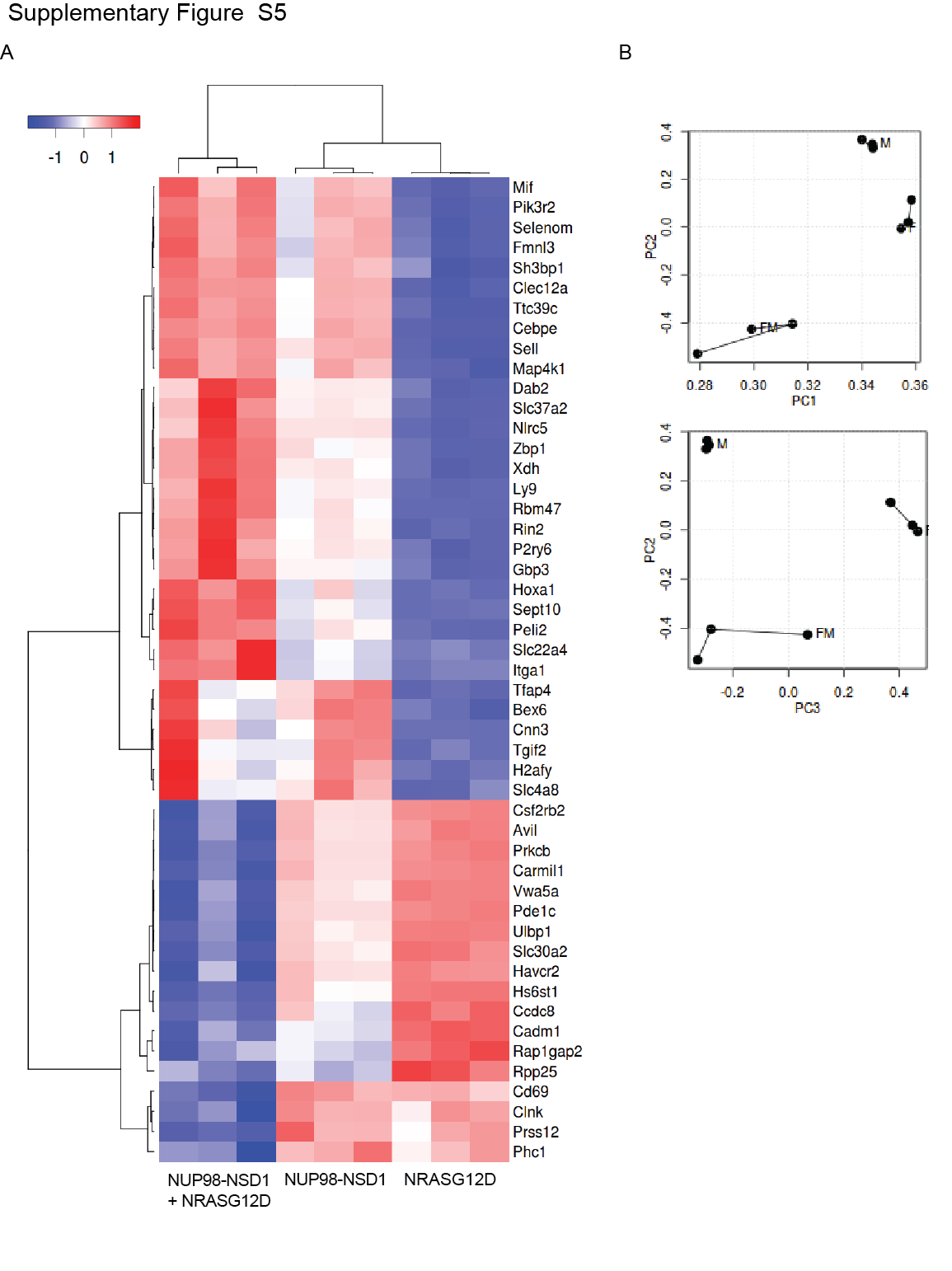


**Supplementary Figure S5.** **Gene expression analysis of RNAseq data from in vitro expanded NRASG12D, NUP98-NSD1 and NUP98-NSD1+NRASG12D transduced mouse bone marrow cells.**

1. Heatmap from unsupervised hierarchical clustering showing the top 50 differentially expressed genes among NUP98-NSD1+NRASG12D**,** NUP98-NSD1 and NRASG12D transduced mouse bone marrow cells. (N=3)
2. Principal component analysis showed distinct overall gene expression levels in NUP98-NSD1 (F), NRASG12D (M) and the combination of NUP98-NSD1+NRASG12D (FM) cells. (N=3)

**
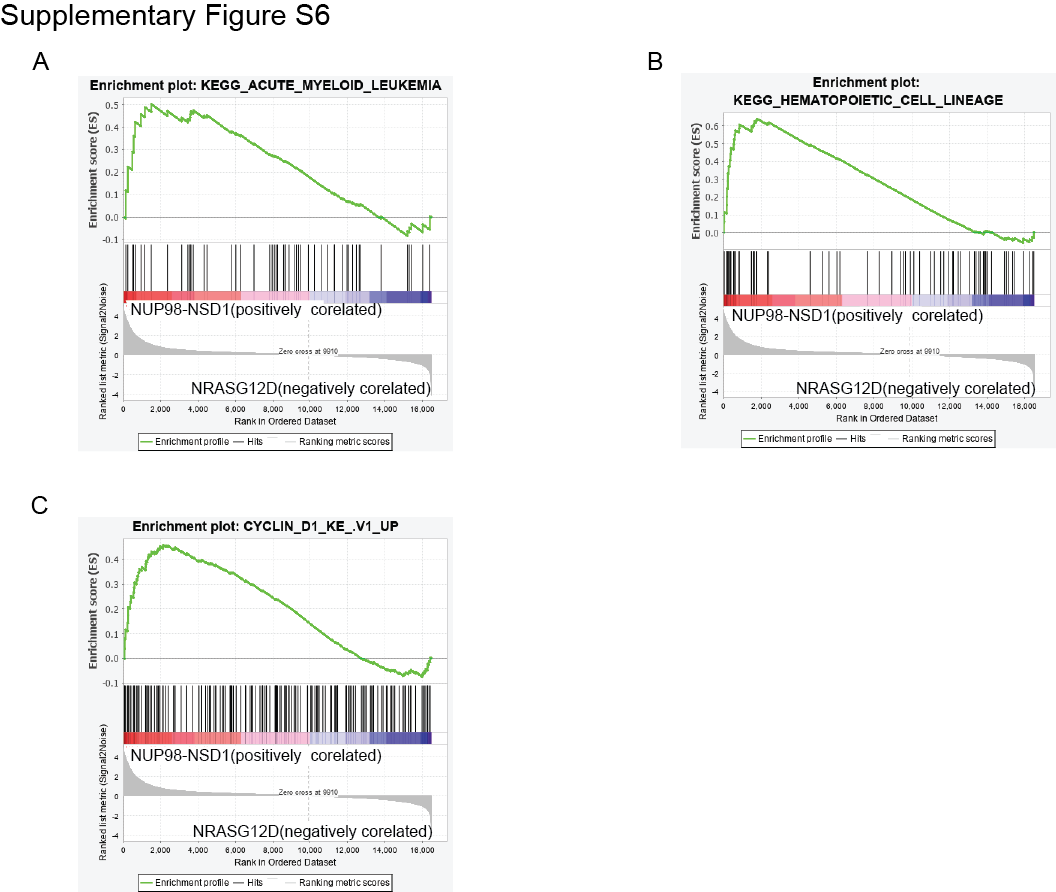
**

**Supplementary Figure S6.** **Gene set enrichment analysis of NUP98-NSD1 transduced mouse bone marrow cells (NRASG12D vs NUP98-NSD1) and gene set enrichment analysis of NRASG12D transduced mouse bone marrow cells (NUP98-NSD1+NRASG12D vs NUP98-NSD1)**

1. Enrichment plot for the KEGG pathway Acute Myeloid Leukemia in NUP98-NSD1 compared to NRASG12D cells.
2. Enrichment plot for the KEGG pathway Hematopoietic cell lineage in NUP98-NSD1 compared to NRASG12D cells.
3. Enrichment plot for the gene set oncogenic cyclin D1 in NUP98-NSD1 compared to NRASG12D cells.


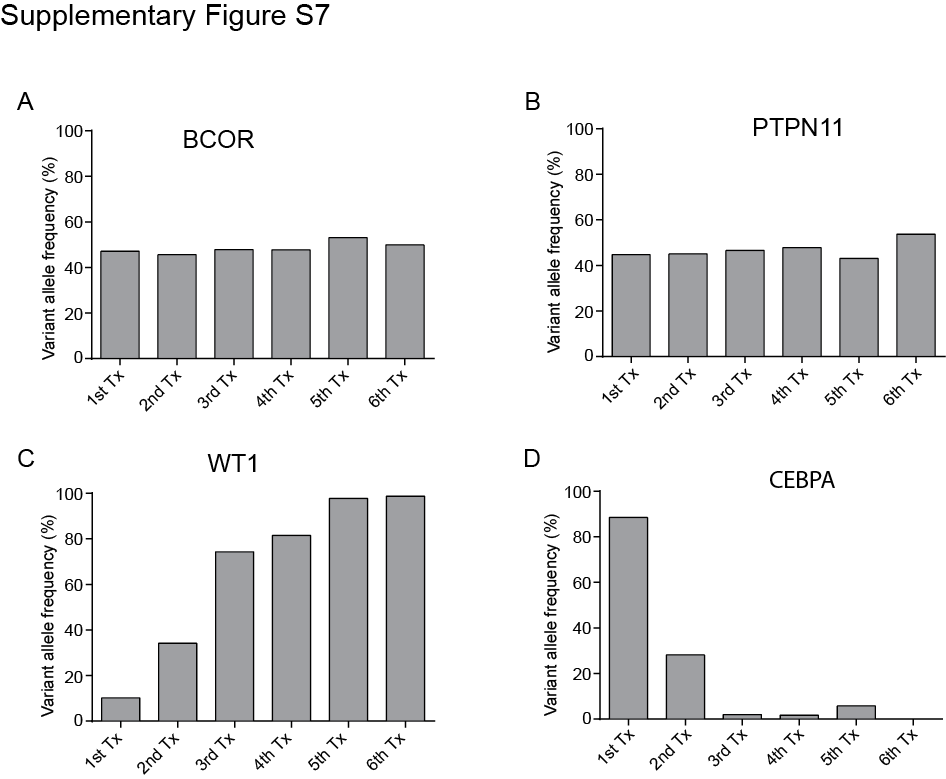


**Supplementary Figure S7.** **Identification and variant allele frequency of mutations in the NUP98-NSD1 PDX model from first to sixth recipient.**

1. Variant allele frequency of the BCOR mutation from first to sixth recipient of serial transplantations of NUP98-NSD1 cells.
2. Variant allele frequency of the PTPN11 mutation from first to sixth recipient of serial transplantations of NUP98-NSD1 cells.
3. Variant allele frequency of the WT1 mutation from first to sixth recipient of serial transplantations of NUP98-NSD1 cells.
4. Variant allele frequency of the CEBPA mutation from first to sixth recipient of serial transplantations of NUP98-NSD1 cells.


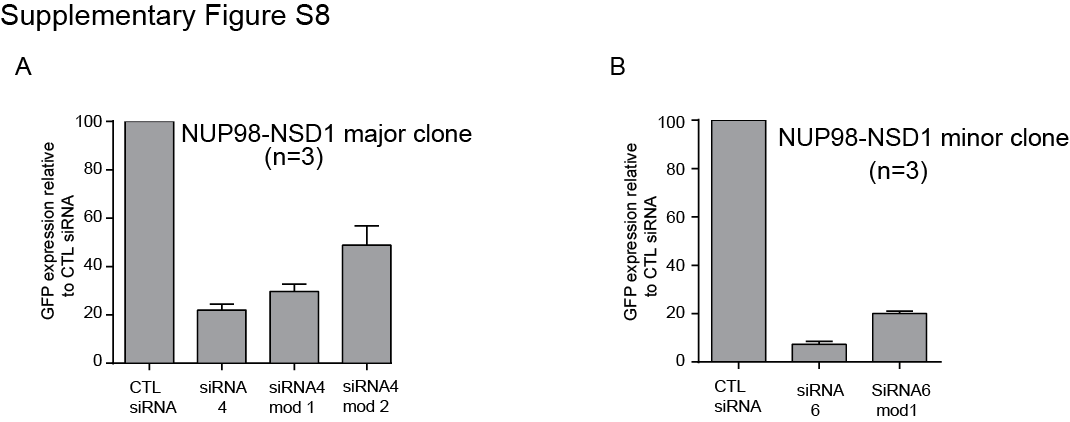


**Supplementary Figure S8.** **Identification and validation of siRNAs against the NUP98-NSD1 fusions.**

1. Knockdown efficacy of siRNA4 with two chemical modifications (mod1, mod2), which are required for the use of the siRNAs *in vivo* (mean ± SEM).
2. Knockdown efficacy of siRNA6 with chemical modifications, which is required for the use of the siRNAs *in vivo* (mean ± SEM).


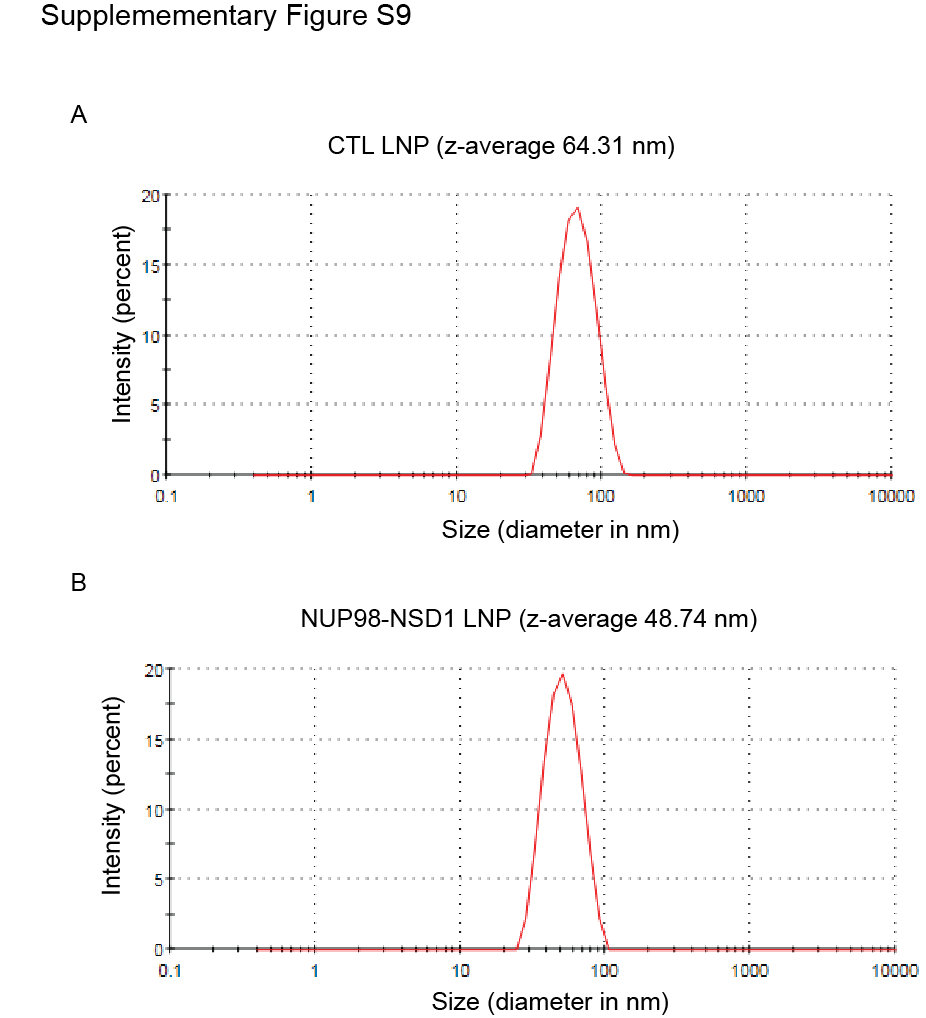


**Supplementary Figure S9.** **Characterization of the siRNA-LNP formulations using the zetasizer.**

1. Representative image of the size distribution of the CTL siRNA-LNPs. Z-Average refers to harmonic intensity averaged particle diameter and the size distribution is based on scattering intensity of each particle fraction.
2. Representative image of the size of anti-NUP98-NSD1 siRNA-LNPs.


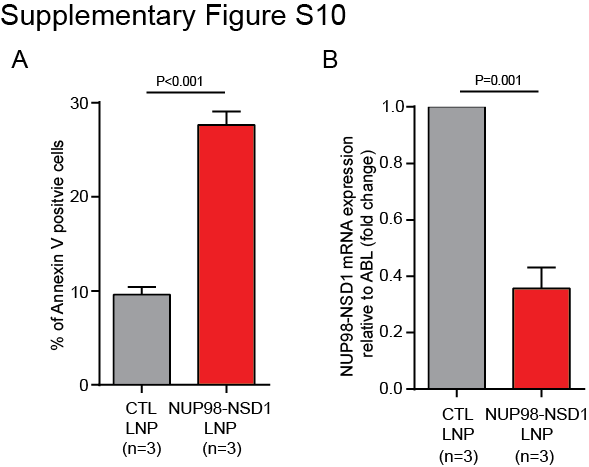


**Supplementary Figure S10.** **Efficacy of siRNA-LNP formulations in *ex vivo* PDX cells.**

1. Percentage of apoptotic cells in CTL LNP and NUP98-NSD1 LNP PDX cells treated ex vivo (mean ± SEM). (concentration 1 μg/ml siRNA on day 0 and day 2).
2. Percentage of knock down of NUP98-NSD1 mRNA in ex vivo treated PDX cells (mean ± SEM). (concentration 1 μg/ml siRNA on day 0 and day 2)


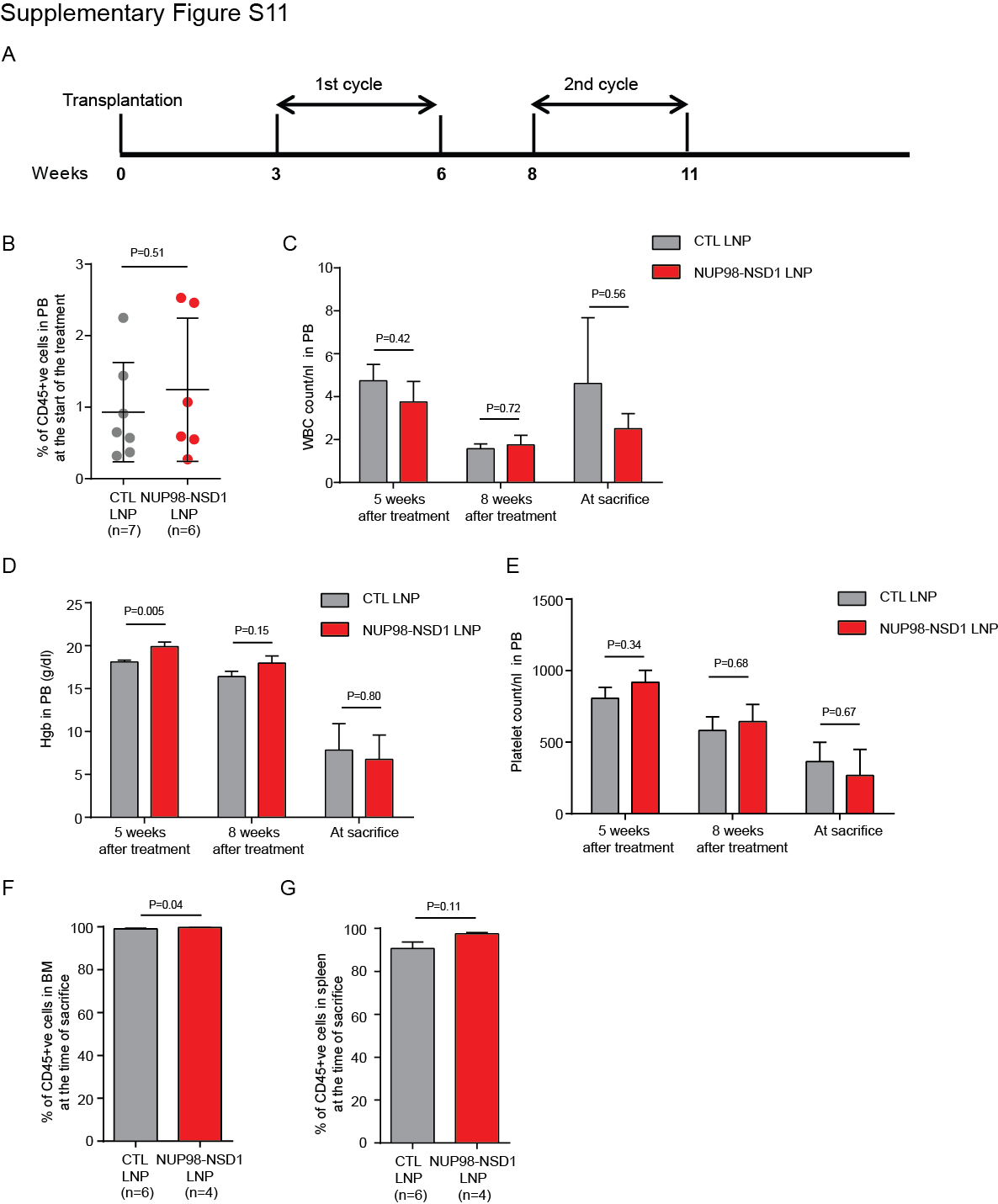


**Supplementary Figure S11.** **Engraftment of leukemic cells and blood counts at different time points during LNP treatment.**

1. Schematic outline of the LNP-siRNA treatment schedule in the NUP98-NSD1 PDX model.
2. Engraftment of human CD45-positive AML cells at the start of the treatment in peripheral blood of NUP98-NSD1 AML PDX mice treated with control or NUP98-NSD1 siRNA/LNP formulations (mean ± SEM).
3. White blood cell count in peripheral blood at different time points of NUP98-NSD1 AML PDX mice treated with control or NUP98-NSD1 siRNA/LNP formulations (mean ± SEM).
4. Hemoglobin levels in peripheral blood at different time points of NUP98-NSD1 AML PDX mice treated with control or NUP98-NSD1 siRNA/LNP formulations (mean ± SEM).
5. Platelet count in peripheral blood at different time points of NUP98-NSD1 AML PDX mice treated with control or NUP98-NSD1 siRNA/LNP formulations (mean ± SEM).
6. Engraftment of human CD45-positive AML cells at the time of sacrifice in bone marrow of NUP98-NSD1 AML PDX mice treated with control or NUP98-NSD1 siRNA/LNP formulations (mean ± SEM).
7. Engraftment of human CD45-positive AML cells at the time of sacrifice in spleen of NUP98-NSD1 AML PDX mice treated with control or NUP98-NSD1 siRNA/LNP formulations (mean ± SEM).

**References**

1. Valtieri M, Schiro R, Chelucci C, Masella B, Testa U, Casella I, et al. Efficient transfer of selectable and membrane reporter genes in hematopoietic progenitor and stem cells purified from human peripheral blood. Cancer research. 1994;54(16):4398-404.
